# The effects of approach bias modification on smoking cue-reactivity in individuals who smoke: A randomized controlled fMRI study

**DOI:** 10.1038/s41598-026-45748-y

**Published:** 2026-03-28

**Authors:** Franziska Motka, Haoye Tan, Sabine Vollstädt-Klein, Katja Bertsch, Charlotte E. Wittekind

**Affiliations:** 1 Division of Clinical Psychology and Psychological Treatment, Department of Psychology, Leopoldstraße 13, 80802 Munich, Germany; 2 NeuroImaging Core Unit Munich (NICUM), University Hospital, LMU Munich, Munich, Germany; 3 Central Institute of Mental Health, University of Heidelberg, Mannheim, Germany; 4 Mannheim Center of Translational Neurosciences (MCTN), Medical Faculty of Mannheim, University of Heidelberg, Mannheim, Germany; 5 German Center of Mental Health (DZPG), Partner Site Mannheim-Heidelberg-Ulm, Mannheim, Germany; 6 Department of Psychology I (Biological Psychology, Clinical Psychology, and Psychotherapy), University of Würzburg, Würzburg, Germany; 7 German Center for Mental Health (DZPG), Partner Site Munich/Augsburg, Munich, Germany

**Keywords:** approach bias modification, smoking, cue-reactivity, fMRI, addiction, smoking cessation, Diseases, Medical research, Neuroscience

## Abstract

Approach bias modification (ApBM), a computerized training designed to retrain involuntary approach action tendencies toward drug-related cues, has been shown to reduce relapse rates when added to treatment-as-usual (TAU) in alcohol use disorder. A potential working mechanism involves reduced neural drug cue-reactivity in reward-related brain regions. In smoking cessation, however, the efficacy and neural mechanisms of ApBM remain unclear. In this randomized-controlled trial, individuals with chronic, moderate-to-heavy tobacco dependence (*N* = 117, *M*_age _= 41.5, 45.3% female) received a one-day smoking cessation intervention (TAU) and were subsequently randomized to complete seven sessions of ApBM (TAU+ApBM), Sham control training (TAU+Sham), or no training (TAU-only). Neural reactivity toward smoking-related versus neutral stimuli (smoking cue-reactivity) was assessed using functional magnetic resonance imaging (fMRI) before and after intervention. Abstinence was the primary clinical outcome. Results showed no significant group×time interactions on cue-reactivity, and ApBM did not enhance abstinence rates. In the precuneus (sensorimotor region), *increased* cue-reactivity following ApBM was associated with higher long-term abstinence probability, while the control groups showed the opposite descriptive pattern. In conclusion, ApBM did not reduce smoking cue-reactivity in reward-related regions, consistent with the lack of beneficial effects on clinical outcomes. Alternative neural target processes (e.g., sensorimotor-related) and respective training procedures should be explored.

**Supplementary Information:**

The online version contains supplementary material available at 10.1038/s41598-026-45748-y.

## Introduction

Tobacco smoking remains a leading cause of morbidity and premature death worldwide, with substantial economic costs^1^. Despite the availability of evidence-based treatments, relapse rates remain high^2,3^. More recent approaches focus on modifying unconscious appetitive responses toward drug-related stimuli, such as approach biases—the automatic tendency to approach drug cues. These responses are believed to play a key role in the development and maintenance of substance use disorders. Specifically, problematic drug use is thought to be driven by strong cue-triggered appetitive responses that are insufficiently regulated^4^. Neurobiologically, these responses are believed to be associated with hyperactivation of mesocorticolimbic reward circuit after prolonged drug use, attributing incentive salience toward drug cues^5^. Neuroimaging studies in smoking support this assumption, showing heightened activity in mesocorticolimbic regions (e.g., striatum, amygdala) in response to smoking-related versus neutral stimuli (smoking cue-reactivity)^6,7^. Importantly, smoking cue-reactivity has been shown to predict relapse^8,9^, highlighting its clinical relevance for smoking cessation.

Approach bias modification (ApBM) aims to “re-train” approach biases by requiring individuals to consistently avoid (e.g., push away) drug-related stimuli in computerized training^10^. In alcohol use disorder, ApBM has shown to significantly reduced both early^11,12^ and long-term relapse rates^13,14^ when added to treatment-as-usual (TAU; e.g., detoxification). These findings have led to its inclusion in treatment guidelines for alcohol use disorder in Germany^15^ and Australia^16^(see R. W. Wiers et al.^17^ for a review). However, evidence that reductions in behavioral approach biases mediate the effects of ApBM on clinical outcomes remains limited^17,18^. Beyond behavioral mechanisms, a functional magnetic resonance imaging (fMRI) study examined ApBM effects on neural activity in abstinent inpatients with alcohol use disorder. Compared with Sham control training (i.e., training requiring individuals to approach and avoid drug-related stimuli with equal probability), ApBM reduced alcohol cue-reactivity in the amygdala, which correlated with decreased craving. These findings suggest that ApBM reduces neural drug cue-reactivity within key regions of the mesocorticolimbic reward circuit^19^.

Compared to alcohol studies, evidence for the efficacy of ApBM in smoking cessation is mixed. While some studies report reductions in cigarette consumption^20,21^ and higher 3-month abstinence rates^22^, others found no significant effects^23–25^. Furthermore, findings challenge the assumption that clinical effects are mediated by changes in behavioral approach biases^26^. To better understand its mechanisms, further research is needed to investigate *how* ApBM might influence responses toward smoking-related stimuli beyond behavioral approach biases. No study has yet examined its effects on neural smoking cue-reactivity—a gap the current fMRI study aims to address. Specifically, we investigated the effects of adding ApBM to TAU on neural smoking cue-reactivity compared to Sham training and TAU-only in adults with chronic, moderate-to-heavy tobacco dependence. We hypothesized that: (1) ApBM would lead to greater reductions in neural reactivity toward smoking-related versus neutral stimuli (smoking cue-reactivity) in reward-related brain regions (e.g., striatum, amygdala); (2) reductions in cue-reactivity would predict a higher probability for short- (post-intervention) and long-term abstinence (6-month follow-up); and (3) cue-reactivity would be associated with craving and behavioral approach biases.

## Methods and materials

### Study overview

**Fig. 1:**
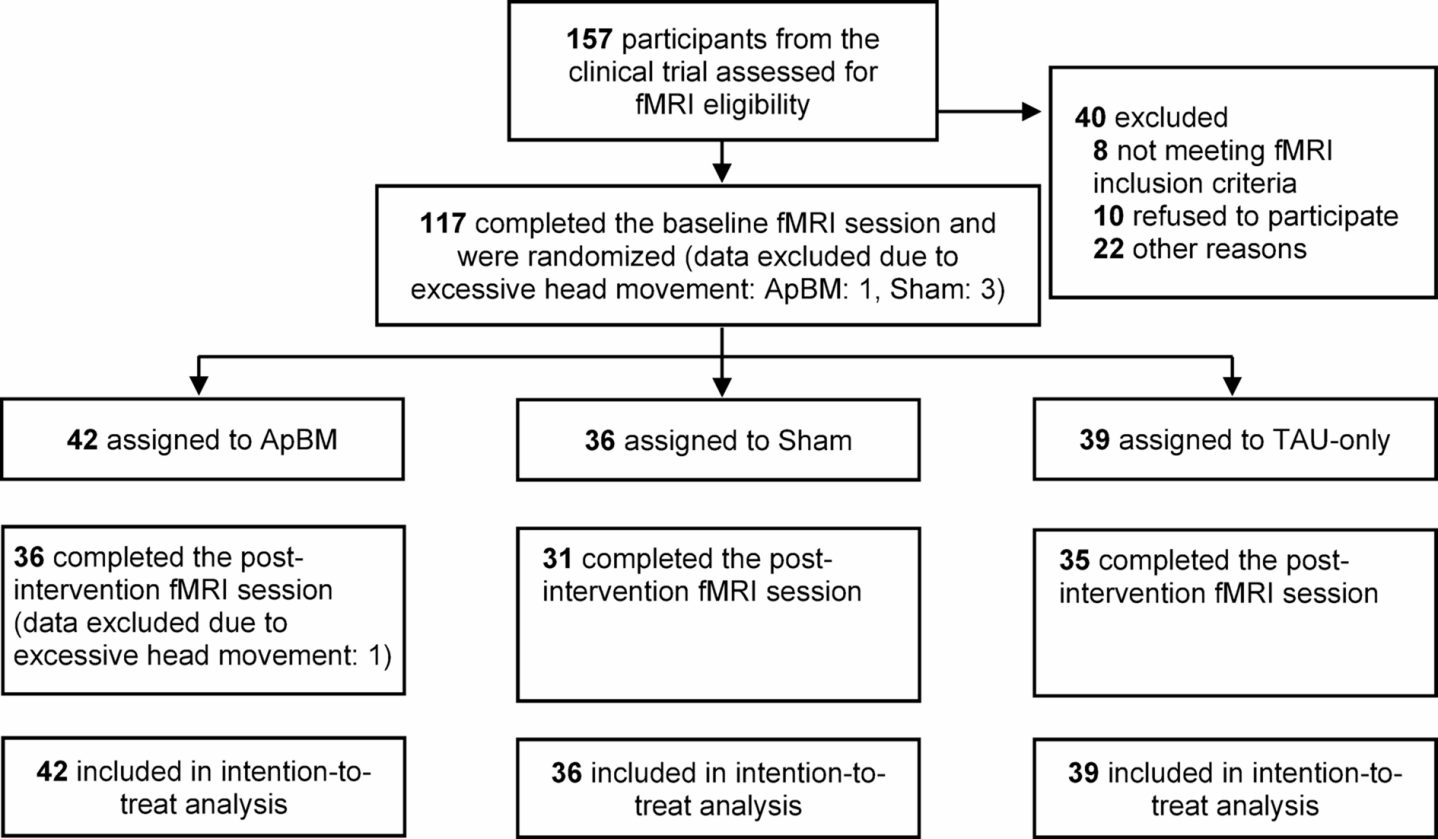
Flow of participants. *Note*. All individuals (*n* = 157) participating in the clinical trial from March 2022 (when the functional magnetic resonance imaging [fMRI] assessments began) were invited to take part in the optional fMRI investigation until recruitment for the clinical trial concluded in March 2023. TAU = Treatment-as-usual; ApBM = Approach bias modification.

**Fig. 2:**
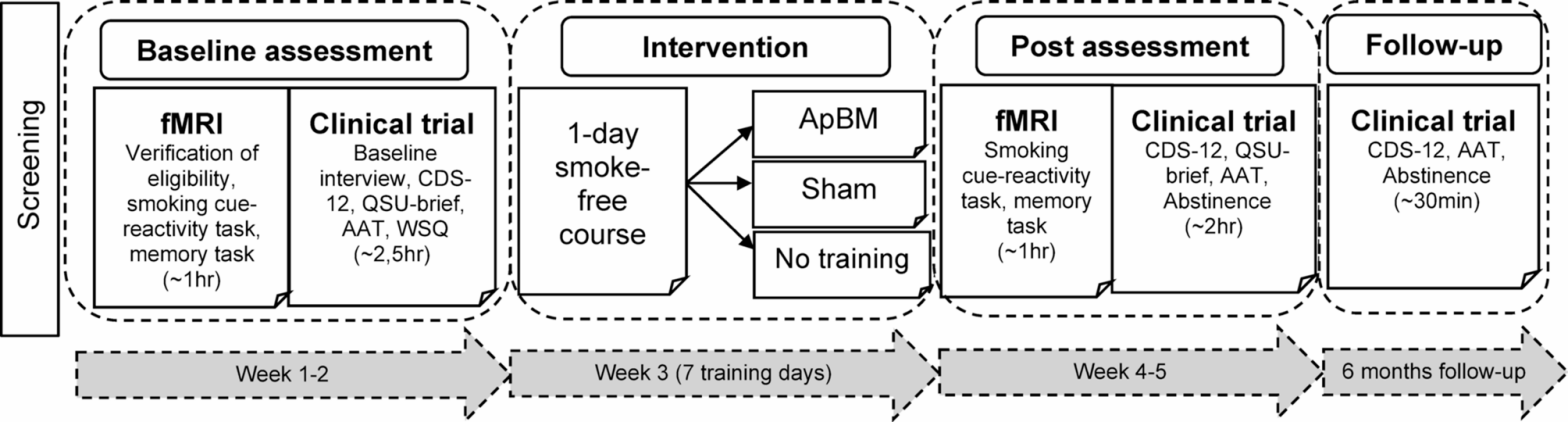
Study procedure.* Note*. This figure illustrates only the questionnaires and tasks relevant to the current functional magnetic resonance imaging (fMRI) investigation. For a complete overview of all assessments conducted in the clinical trial, see Wittekind et al.^26^. CDS-12 = Cigarette Dependence Scale, 12-item version; QSU-brief = Questionnaire on Smoking Urges, brief version; AAT = Approach-Avoidance task; WSQ = Web Screening Questionnaire; ApBM = Approach bias modification.

This fMRI study was part of a preregistered, randomized-controlled, double-blind, single-center, parallel-group superiority trial on the efficacy of ApBM as an add-on to smoking cessation treatment (German Clinical Trials Register: DRKS00019221 [https://drks.de/search/en/trial/DRKS00019221], 11/11/2019; for the study protocol, see Wittekind et al.^26^). The clinical trial included participants from November 2019 to March 2023, with the fMRI substudy enrolling *N* = 117 participants (see Fig. 1 [participant flow]) between March 2022 and March 2023. All participants completed a smoke-free course with quit attempt (TAU) before randomization (1:1:1) to one of three arms: (1) TAU+ApBM, (2) TAU+Sham, or (3) TAU-only (see Fig. 2). The study was approved by the ethics committee of Ludwig-Maximilians-University (LMU) Munich (23_Wittekind_c_2019) and conducted in accordance with the Declaration of Helsinki and relevant institutional and national guidelines. All participants provided written informed consent after being fully informed about the study purpose, procedures, and potential risks and benefits.

### Participants and sensitivity analysis

Participants were recruited by various means, including social media ads, online marketing, flyers distributed in medical practices and universities, and newsletters. Clinical trial inclusion criteria were: (1) age 18–70 years, (2) Fagerström Test for Nicotine Dependence (FTND)^27^ score ≥ 3 (moderate-to-heavy dependence)^28^, (3) exhaled carbon monoxide (CO) ≥ 10ppm, (4) smoking ≥ 10 cigarettes daily within the past 12 months, (5) willingness to abstain from any smoking cessation interventions (e.g., e-cigarettes) during the study, and (6) motivation to participate in the smoking cessation intervention. Exclusion criteria were: (1) current/past diagnosis of severe psychiatric (bipolar disorder, psychosis) or major neurological disorders (multiple sclerosis, Parkinson’s disease), (2) moderate or severe substance use disorder (≥ 4 DSM-5 criteria, as assessed with the Mini International Neuropsychiatric Interview [MINI]^29^ other than tobacco within the past 12 months, (3) use of nicotine replacement therapy or pharmacological smoking cessation treatments within three months prior to study participation, (4) acute suicidality, (5) current pregnancy or nursing period, and (6) insufficient German language skills. Further fMRI-specific exclusion criteria were: (1) standard MRI contraindications (e.g., pacemaker), (2) psychotropic medication use, (3) head trauma history, and (4) uncorrectable visual impairments. Participants received either financial compensation or course credit for assessment participation (clinical trial: 8€/hour; fMRI: 25€ at baseline, 35€ at post-intervention).

A sensitivity analysis was conducted using G*Power^30^. Assuming *α* = 0.05 and 1-*β* = 0.80 with a sample size of *N* = 117, the minimum detectable effect size was *f* = 0.145 (*d* = 0.290; small-to-medium^31^. This effect size is substantially smaller than the between-group effect reported by C. E. Wiers et al.^19^ for the effect of ApBM versus Sham training on alcohol cue-reactivity in the left amygdala (*d* = 1.08).

### Study procedure, randomization, and blinding

Individuals interested in the clinical trial completed a telephone screening to assess eligibility, explain procedures, offer the optional fMRI investigation, and schedule appointments. Baseline (t_0_) assessments for the fMRI and clinical trial study were conducted separately (fMRI: Neuroimaging Core Unit Munich [NICUM], LMU; clinical trial: laboratory of the Department of Psychology, LMU) within two weeks before the smoke-free course, with the fMRI session always scheduled first (see Fig. 2). Inclusion and exclusion criteria were verified before the fMRI session. Participants were reassessed after the intervention phase (t_1_: within a two-week timeframe) and at the 6-month follow-up (t_2_; within a four-week timeframe).

The study used block-wise randomization, assigning all participants of a given smoke-free course to the same arm. The randomization sequence was generated externally (Munich Centre of Clinical Trials). A staff member not involved in recruitment and assessments informed participants of their group assignment after the smoke-free course, conducted the first training session, monitored compliance, and sent reminders. Other staff (e.g., assessors, smoke-free trainers) remained blinded. Participants in the ApBM and Sham groups were blinded to training condition. Blinding was not possible for the TAU-only group.

## Interventions

### Smoking cessation intervention (TAU)

The cessation intervention included a one-day (total duration: 6 h), manualized cognitive-behavioral group intervention (smoke-free course), led by certified trainers. No preparatory intervention sessions were conducted prior to the course, and the use of nicotine replacement therapy or other pharmacological smoking cessation treatments was not permitted during study participation (see exclusion criteria). An optional 15-minute telephone counseling session was offered one week later. The course comprised four sections integrating psychoeducation, motivational interviewing, cognitive strategies, goal-oriented techniques, and a collective quit attempt^32^. More details are provided in the supplementary methods. Participants in the fMRI study were recruited from 12 smoke-free courses, with an average of 13 participants per course.

### Study Arms: Approach bias modification, sham training, and TAU-only

Participants in the ApBM and Sham training groups were instructed to complete seven daily sessions using a joystick on their home computer. The first session was conducted immediately after the smoke-free course. Each session began with ten practice trials, followed by 240 training trials including 20 smoking-related pictures (e.g., burning cigarettes; simple, context-free pictures, consistent with previous alcohol ApBM studies^13,33,34^ and 20 positive pictures (e.g., men fishing). In each trial, participants were instructed to push or pull the joystick based on picture tilt (5° left/right), with instruction (pull vs. push right-tilted pictures) counterbalanced across participants. To simulate approach/avoidance, pictures increased in size when pulled and decreased when pushed. In ApBM training, smoking-related pictures were consistently pushed (avoidance) and positive pictures pulled (approach); in Sham training, assignments were balanced. Training was implemented in Inquisit^®^. TAU-only participants received no additional training.

### Data collection

#### Interviews, questionnaires, and behavioral tasks

Sociodemographic and smoking-related data (e.g., cigarettes per day, smoking duration) were collected during the clinical trial baseline assessment. Tobacco dependence severity was measured using the 12-item Cigarette Dependence Scale-12 (CDS-12)^35^, aligning with DSM-IV and ICD-10 criteria. Mental health disorders were screened with the Web Screening Questionnaire (WSQ)^36^. The primary clinical outcome, abstinence at t_2_, was defined according to the Russell Standard criteria^37^(a maximum of five cigarettes smoked until follow-up; biochemical verification; smoking status classified as smoking, if not certifiably verified; analysis of participants with protocol violations; and blinded follow-up data collection), with abstinence also assessed at t_1_. Intervention effects on behavioral approach biases were assessed using the joystick-based Approach-Avoidance Task^38^(AAT; see supplementary methods for task details). Of the 40 smoking-related and 40 positive AAT pictures, half overlapped with ApBM/Sham training and half were novel (untrained) to test generalization.

### Neural smoking cue-reactivity task

**Fig. 3:**
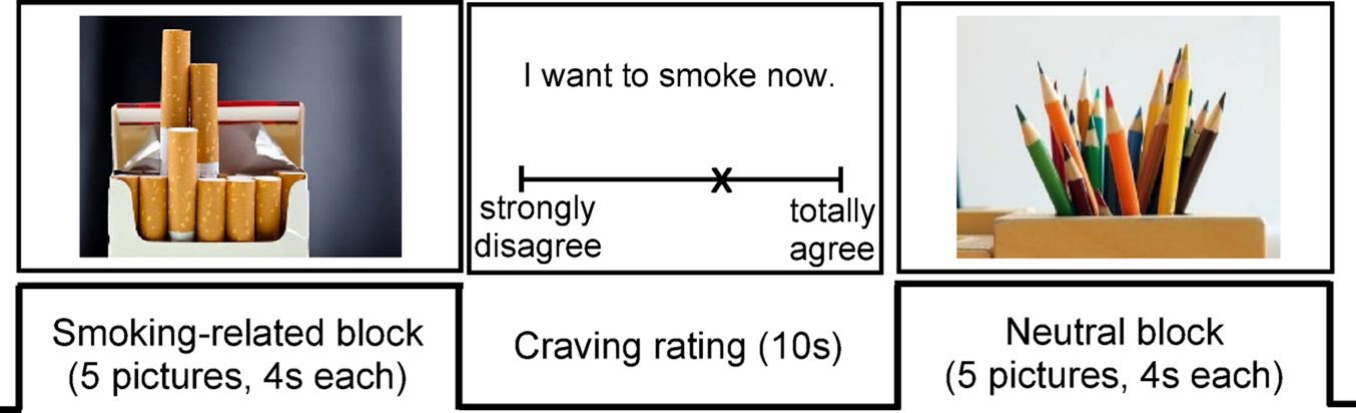
Neural smoking cue-reactivity paradigm. Note. The smoking cue-reactivity paradigm comprised 40 smoking-related and 40 shape- and color-matched neutral stimuli, presented in eight blocks per category in randomized order. Block categories followed a fixed sequence, with no more than two consecutive blocks from the same category (s-s-n-s-n-s-s-n-s-n-s-n-n-s-n-n; s = smoking-related, n = neutral). Each block contained five stimuli, each displayed for 4 seconds. After each block, participants rated their current desire to smoke (“*I want to smoke now.*”) on a visual analog scale from 0 (“*strongly disagree*”) to 100 (“*totally agree*”) within 10 seconds using a button press.

Neural reactivity toward smoking-related stimuli was assessed with an adapted alcohol cue-reactivity paradigm^39^(see Fig. 3). Stimuli included the 40 smoking-related AAT pictures and 40 shape- and color-matched neutral pictures (e.g., pencils), randomly presented across eight smoking-related and eight neutral blocks (five stimuli per block presented for 4 s each). The block order was fixed, with no more than two consecutive blocks from the same category. Participants passively viewed the stimuli and rated smoking desire (“*I want to smoke now.*”) after each block on a visual analog scale from 0 (“*strongly disagree*”) to 100 (“*totally agree*”) within 10 s using a button press. A black fixation cross then appeared for at least 10 s, ensuring a total duration of 20 s for craving rating and fixation. The task lasted approximately 10 min (total scan duration: 30 min). Post-scan, attention was assessed via an unannounced recognition task requiring classification of 10 previously presented and 10 new pictures as “*known*” or “*unknown*”. fMRI data acquisition details are given in the supplementary methods.

### Data analysis

#### Data preprocessing and measure extraction

##### Behavioral data

AAT data were preprocessed and analyzed using R (version 4.3.0; R Core Team, 2023). Details on the preprocessing procedure are provided in the supplementary methods. As preregistered (see Wittekind et al.^26^), the AAT effect score was calculated by subtracting the median RT of final push movements (RT from picture onset to full joystick extension) from that for pull movements in trials with untrained smoking-related stimuli (push_smoking _– pull_smoking_). Higher scores indicate a stronger approach bias towards smoking-related stimuli.

### Neuroimaging data

Neuroimaging data were preprocessed using SPM12 (https://www.fil.ion.ucl.ac.uk/spm) in MATLAB R2023a (The Mathworks, Natick, MA, USA). The SPM12 pipeline included spatial realignment, co-registration, normalization to a standard 2 mm Montreal Neurological Institute [MNI] template, and spatial smoothing (Gaussian kernel, 8 mm full width at half maximum [FWHM]). Given the task block design, slice-timing correction was not applied. fMRI data of participants with excessive head movement at t_0_ or t_1_ (> 3 mm in any direction or > 3° rotation) were excluded from analysis (*n* = 5; see Fig. 1). Individual-level statistical analysis was conducted using a general linear model (GLM) with the three task block conditions (smoking-related/neutral/rating) as regressors, modeled as boxcar functions convolved with the hemodynamic response function. Motion parameters and a constant term were included as nuisance regressors, and a high-pass filter (1/128 Hz) was applied.

Both whole-brain and hypothesis-driven region of interest (ROI) analyses were employed at the group level using SPM12. To identify brain regions showing greater blood oxygenation level-dependence (BOLD) responses in smoking-related versus neutral blocks at t_0_, whole-brain one-sample *t*-tests on the contrast [smoking – neutral] were performed. To examine changes in smoking cue-reactivity from t_0_ to t_1_ between groups, an ANOVA on the contrast (pre [smoking – neutral] – post [smoking – neutral]) was conducted with group as between-subject factor. Both analyses were controlled for age and sex. For the ROI analysis, the left anterior cingulate cortex, left angular gyrus, right thalamus, dorsal striatum, nucleus accumbens, amygdala, and medial prefrontal cortex were selected based on prior research on smoking cue-reactivity^6^, as well as on neural correlates of alcohol approach biases^40^ and effects of alcohol ApBM^19^. The medial prefrontal cortex mask was taken from the Harvard-Oxford probabilistic atlas (mask: MedFC)^41–44^, while all other masks were generated using the Automated Anatomical Labeling atlas (AAL3)^45^. Mean values for the contrast [smoking – neutral] at t_0_ and t_1_ were extracted for each ROI. Visual attention during the cue-reactivity task can be considered high, with approximately 82% of stimuli at t_0_ and t_1_ correctly classified as “*known*” or “*unknown*”.

### Statistical analysis

Analyses followed an intention-to-treat (ITT) approach in accordance with CONSORT guidelines^46^. Cue-reactivity at t_0_ was assessed by testing the mean [smoking – neutral] contrast against zero in each ROI using parametric one-sample *t*-tests with Cohen’s *d*^31^ or non-parametric Wilcoxon signed-rank test with Rosenthal’s *r*^47^. Test selection was guided by Shapiro-Wilk tests of normality. Intervention effects on cue-reactivity in each ROI (Hypothesis 1) were examined using linear mixed-effects models (LMMs; *lme4* package^48^ incorporating all available data^49^. Outcome measures were modeled with dummy-coded time (0: t_0_; 1: t_1_), group (0: TAU-only; 1: TAU+Sham; 2: TAU+ApBM), and the time×group interaction (i.e., between-group differences in pre-post change) as predictors, with participant- and course-level random intercepts. Analyses were repeated for participants with high training adherence (≥ 5 sessions; preregistered criterion, see Wittekind et al.^26^). Associations between changes in cue-reactivity and abstinence at t_1_ and t_2_ (Hypothesis 2) were assessed using logistic regressions with abstinence predicted by cue-reactivity change, group, and their interaction. Associations between cue-reactivity and behavioral variables (AAT effect scores, craving ratings; Hypothesis 3) were examined using LMMs with changes in behavioral variables predicted by cue-reactivity change, group, and their interaction. Supplementary analyses also assessed the association between cue-reactivity changes and smoking-related variables (cigarettes per day, tobacco dependence severity, CO value).

Across analyses, main and interaction effects were evaluated using omnibus tests (analysis of variance [ANOVA]; *Anova* function). Significant effects were followed up using the *emmeans* package^50^ to obtain estimated marginal trends. All statistical analyses were conducted using an *α*-level of 0.05 and two-tailed tests. To control the false discovery rate (FDR) at 5%, we applied the Benjamini-Hochberg correction^51^ for multiple comparisons across ROIs for omnibus main and interaction effects separately. Measure reliability was estimated using Cronbach’s α for questionnaires and split-half reliability for the AAT effect score and neural cue-reactivity ([smoking – neutral] contrast) using the *splithalfr* package^52^ with 5,000 random splits and Spearman-Brown correction.

## Results

### Sample characteristics and adherence

Sociodemographic and clinical characteristics are depicted in Table 1. Participants showed a chronic smoking history (*M* = 22.81 years, *SD* = 11.19) and moderate-to-heavy tobacco dependence (cigarettes per day: *M* = 18.23, *SD* = 5.97; FTND score: *M* = 5.33, *SD* = 1.63). No significant baseline differences between groups emerged. Retention rates at t_1_ (TAU+ApBM: 85.7%, TAU+Sham: 86.1%, TAU-only: 89.7%; see Fig. 1) and training adherence were high (84.7% completed at least five training sessions; see Table 1).

**Table 1:** *Baseline characteristics and training adherence*.

Variables	Whole sample (*N* = 117)	TAU+ApBM (*n* = 42)	TAU+Sham (*n* = 36)	TAU-only (*n* = 39)	Tests for group differences (*p*-value)
Sociodemographic information					
Age in years	41.47 (11.67)	42.83 (11.89)	41.58 (10.35)	39.90 (12.65)	0.530
Sex [*n*, (% female)]	53 (45.3)	19 (45.2)	15 (41.7)	19 (48.7)	0.829
German university entrance qualification “Abitur” [*n*, (%)]	68 (58.1)^2^	28 (66.7)^2^	17 (47.2)	23 (59.0)	0.173
Clinical characteristics					
Cigarettes per day	17.96 (5.99)	18.26 (5.08)	18.00 (6.78)	17.59 (6.25)	0.881
Smoking duration in years	22.81 (11.19)^1^	23.77 (11.41)	22.90 (10.23)^2^	21.71 (11.93)	0.710
CDS-12 (scale range: 0–48)	35.41 (5.59)	36.31 (5.22)	34.64 (5.41)	35.15 (6.12)	0.399
FTND (scale range: 0–10)	5.33 (1.63)	5.38 (1.45)	5.58 (1.75)	5.05 (1.70)	0.362
CO value	33.47 (17.25)	34.57 (16.73)	31.89 (15.54)	33.74 (19.49)	0.788
Psychiatric disorders^2^ [n, (%)]	93 (79.5)	33 (78.6)	30 (83.3)	30 (76.9)	0.777
Training adherence (*N* = 72)^3^					
Mean number of absolved training sessions	7.06 (2.57)	7.41 (2.34)	6.64 (2.79)	–	0.205
Number of participants who completed ≥ 5 training sessions [n, (%)]	61 (84.7)	35 (89.7)	26 (78.8)	–	0.338

Note. Values are presented as *M* (*SD*) unless otherwise indicated. Baseline group differences were analyzed using ANOVAs or *t*-tests (ApBM versus Sham training group for adherence variables) for continuous variables and chi-square or Fisher’s exact tests for categorical variables. TAU = Treatment-as-usual; ApBM = Approach bias modification; CDS-12 = Cigarette dependence scale, 12-item version; FTND = Fagerström test for nicotine dependence; CO = Carbon monoxide.

^1^ *n* = 1 missing value.

^2^ Operationalized as exceeding the cut-off score on the Web Screening Questionnaire (WSQ) for at least one of the following disorders: depression, generalized anxiety disorder, panic disorder, agoraphobia, panic disorder with agoraphobia, specific phobia, social phobia, post-traumatic stress disorder, obsessive-compulsive disorder, and alcohol abuse/dependence.

^3^ Six participants (TAU+ApBM: *n* = 3; TAU+Sham: *n* = 3) had missing values, as they did not receive training due to non-attendance at the smoke-free course (TAU).

### Smoking cue-reactivity at baseline

#### Whole-brain analysis

Whole-brain one-sample *t*-tests on the contrast [smoking > neutral] at t_0_ identified three clusters surviving a voxel-wise threshold of *p *< .001 and an extent threshold of FWEc = 336 voxels (*p*_FWE _< 0.05): right middle cingulate cortex (cluster size = 782; MNI coordinates: X = 6, Y = -26, Z = 30; *t*_max _= 5.69, *p*_FWE−corr _< 0.001), right precuneus (cluster size = 336; MNI coordinates: X = 16, Y = -70, Z = 48; *t*_max _= 4.83, *p*_FWE−corr _= 0.023), and right supramarginal gyrus (cluster size = 381; MNI coordinates: X = 62, Y = -20, Z = 32; *t*_max _= 4.39, *p*_FWE−corr _= 0.014). These regions were included in subsequent intervention effect analyses.

### Region of interest analysis

At t_0_, the contrast [smoking – neutral] was significantly greater than zero in the left anterior cingulate cortex (*Z* = 4.50, *p *< .001, *p*_FDR _< 0.001, *r* = .42; see Table 2 for descriptive statistics), indicating higher activity in smoking-related versus neutral blocks. Conversely, it was significantly lower than zero in the right thalamus (*t*[112] = -3.42, *p *< .001, *p*_FDR _= 0.002, *d* = 0.32) and dorsal striatum (*Z *= -2.94, *p *= .003, *p*_FDR _= 0.005, *r* = .28), reflecting reduced activity in smoking-related blocks. No significant differences emerged in the left angular gyrus (*t*[112] = -0.65, *p *= .520), amygdala (*t*[112] = 0.184, *p *= .855), nucleus accumbens (*Z* = 1.24, *p *= .215), and medial prefrontal cortex (*Z *= -0.65, *p *= .515), suggesting comparable activity in smoking-related and neutral blocks.

**Table 2:** *Descriptive statistics and results of the ANOVAs (omnibus tests) for the effects of time and time×group on neural and behavioral outcomes*.

Outcome variable	Time	Whole sample (*N* = 117)		TAU+ApBM (*n* = 42)		TAU+Sham (*n* = 36)		TAU-only (*n* = 39)		Effect of predictor
		*M*	*SD*	*M*	*SD*	*M*	*SD*	*M*	*SD*	
L ACC	t_0_	0.079	0.178	0.070	0.153	0.027	0.169	0.132	0.199	Time: *F*(1,105.34) = 8.73, ***p *= .004**, ***p***_**FDR **_**= 0.025** Time×group: *F*(2,109.44) = 0.50, *p *= .609
	t_1_	0.030	0.211	0.071	0.215	-0.012	0.197	0.025	0.216	
L angular gyrus	t_0_	-0.010	0.161	0.004	0.119	-0.044	0.172	0.006	0.186	Time: *F*(1,104.80) = 0.01, *p *= .922 Time×group: *F*(2,108.92) = 0.46, *p *= .630
	t_1_	-0.005	0.215	0.040	0.190	-0.064	0.236	0.001	0.214	
R thalamus	t_0_	-0.047	0.145	-0.038	0.113	-0.078	0.178	-0.028	0.144	Time: *F*(1,105.34) = 0.19, *p *= .663 Time×group: *F*(2,109.44) = 0.36, *p *= .701
	t_1_	-0.084	0.184	-0.042	0.180	-0.145	0.151	-0.072	0.205	
Dorsal striatum	t_0_	-0.029	0.093	-0.026	0.082	-0.053	0.107	-0.010	0.087	Time: *F*(1,105.28) = 0.001, *p *= .970 Time×group: *F*(2,109.40) = 0.66, *p *= .520
	t_1_	-0.029	0.134	0.013	0.133	-0.075	0.100	-0.031	0.150	
R MCC	t_0_	0.091	0.143	0.090	0.120	0.064	0.150	0.113	0.158	Time: *F*(1,105.34) = 7.47, ***p *= .007**, ***p***_**FDR **_**= 0.025** Time×group: *F*(2,109.44) = 0.54, *p *= .583
	t_1_	0.056	0.213	0.104	0.197	-0.012	0.156	0.066	0.257	
R supramar-ginal gyrus	t_0_	0.080	0.179	0.075	0.162	0.064	0.228	0.098	0.149	Time: *F*(1,105.07) = 5.05, ***p *= .027**, ***p***_**FDR **_**= 0.067** Time×group: *F*(2,109.19) = 0.71, *p *= .492
	t_1_	0.008	0.222	0.025	0.219	-0.010	0.228	0.009	0.225	
R precuneus	t_0_	0.094	0.216	0.087	0.186	0.052	0.269	0.136	0.191	Time: *F*(1,104.80) = 8.19, ***p *= .005**, ***p***_**FDR **_**= 0.025** Time×group: *F*(2,108.91) = 0.37, *p *= .693
	t_1_	0.025	0.278	0.035	0.258	0.001	0.264	0.037	0.314	
Amygdala	t_0_	0.003	0.160	-0.003	0.137	-0.008	0.181	0.018	0.167	Time: *F*(1,105.14) = 1.21, *p *= .273 Time×group: *F*(2,109.26) = 0.30, *p *= .742
	t_1_	-0.065	0.202	-0.090	0.232	-0.079	0.160	-0.026	0.202	
Nucleus accumbens	t_0_	0.026	0.199	0.005	0.189	0.010	0.243	0.063	0.163	Time: *F*(1,104.78) = 2.03, *p *= .157 Time×group: *F*(2,107.78) = 1.47, *p *= .235
	t_1_	-0.025	0.230	0.015	0.226	-0.095	0.237	-0.004	0.219	
M prefrontal cortex	t_0_	0.001	0.163	-0.005	0.163	-0.041	0.164	0.043	0.158	Time: *F*(1,106.12) = 1.22, *p *= .271 Time×group: *F*(2,109.16) = 0.43, *p *= .651
	t_1_	-0.037	0.195	-0.028	0.186	-0.090	0.192	< 0.000	0.202	
AAT effect score	t_0_	-19.009	67.119	-31.829	73.054	-7.694	55.437	-15.974	69.833	Time: *F*(1,108.44) = 5.68, ***p *= .019** Time×group: *F*(1,108.93) = 1.92, *p *= .151
	t_1_	-28.854	55.608	-34.865	47.441	-5.045	54.163	-44.500	58.781	
Craving rating	t_0_	54.007	26.006	54.976	22.487	57.844	22.709	49.423	31.738	Time: *F*(1,105.86) = 16.07, ***p *< .001** Time×group: *F*(1,106.74) = 2.46, *p *= .091
	t_1_	29.924	24.437	32.643	21.654	26.447	23.621	30.129	27.992	

Note. L = left; R = right; M = marginal; TAU = Treatment-as-usual; ApBM = Approach bias modification; t_0 _= Baseline; t_1 _= Post-intervention; AAT = Approach-avoidance task; ACC = anterior cingulate cortex; MCC = middle cingulate cort.

### Hypothesis 1: Intervention effects on smoking cue-reactivity

#### Whole-brain analysis

Whole-brain voxel-wise ANOVAs on the contrast (pre [smoking – neutral] – post [smoking – neutral]) across the 98 participants with complete t_0_ and t_1_ data revealed no significant BOLD activity changes surviving a height threshold of *F*(2,93) = 7.45, *p *< .001.

### Region of interest analysis

Contrary to our hypothesis, omnibus tests revealed no significant group×time interactions on smoking cue-reactivity in any ROI (*p*s ≥ 0.235; see Table 2 and supplementary results Fig. S1 for boxplot visualization), indicating comparable changes in cue-reactivity across groups. Results remained unchanged when only participants with ≥ 5 training sessions were considered. However, significant main effects of time emerged in the left anterior cingulate cortex (*p *= .004, *p*_FDR _= 0.025), right middle cingulate cortex (*p *= .007, *p*_FDR _= 0.025), right precuneus (*p *= .005, *p*_FDR _= 0.025), and right supramarginal gyrus (*p *= .027, *p*_FDR _= 0.067), indicating that cue-reactivity decreased from t_0_ to t_1_ across groups in these ROIs. No significant effects of time were found in other ROIs (*p*s ≥ 0.157), suggesting similar cue-reactivity at t_0_ and t_1_.

### Hypothesis 2: Abstinence rates and associations with smoking cue-reactivity changes

Regarding group differences in abstinence rates, no significant effects were found at t_1_ (short-term abstinence; whole sample: 56 of 117 [47.9%], TAU+ApBM: 19 of 42 [45.2%], TAU+Sham: 19 of 36 [52.8%], TAU-only: 18 of 39 [46.2%]; χ²[2] = 0.51, *p *= .775) and at t_2_ (long-term abstinence; whole sample: 26 of 117 [22.2%], TAU+ApBM: 11 of 42 [26.2%], TAU+Sham: 6 of 36 [16.7%], TAU-only: 9 of 39 [23.1%]; χ²[2] = 1.04, *p *= .594).

Contrary to our hypothesis, none of the examined ROIs showed a significant interaction between group and cue-reactivity changes on short-term abstinence (*p*s ≥ 0.053; see supplementary results Table S1). This indicates that the associations between cue-reactivity changes from t_0_ to t_1_ and abstinence at t_1_ were similar between study groups. Likewise, across the whole sample, no significant main effects of cue-reactivity changes on short-term abstinence emerged (*p*s ≥ 0.058), with one exception: an omnibus test revealed a significant association between changes in amygdala cue-reactivity and abstinence at t_1_ (*p *= .041; non-significant after FDR-correction). Estimated marginal trends indicated that greater reductions in amygdala cue-reactivity were marginally associated with a higher probability of abstinence; however, this association did not reach statistical significance (logit-scale slope = 0.80, *95%CI*[-0.80 to 2.40]; the logit-scale slope reflects the estimated change on the log-odds of abstinence associated with a one-unit reduction in amygdala cue-reactivity).

**Fig. 4:**
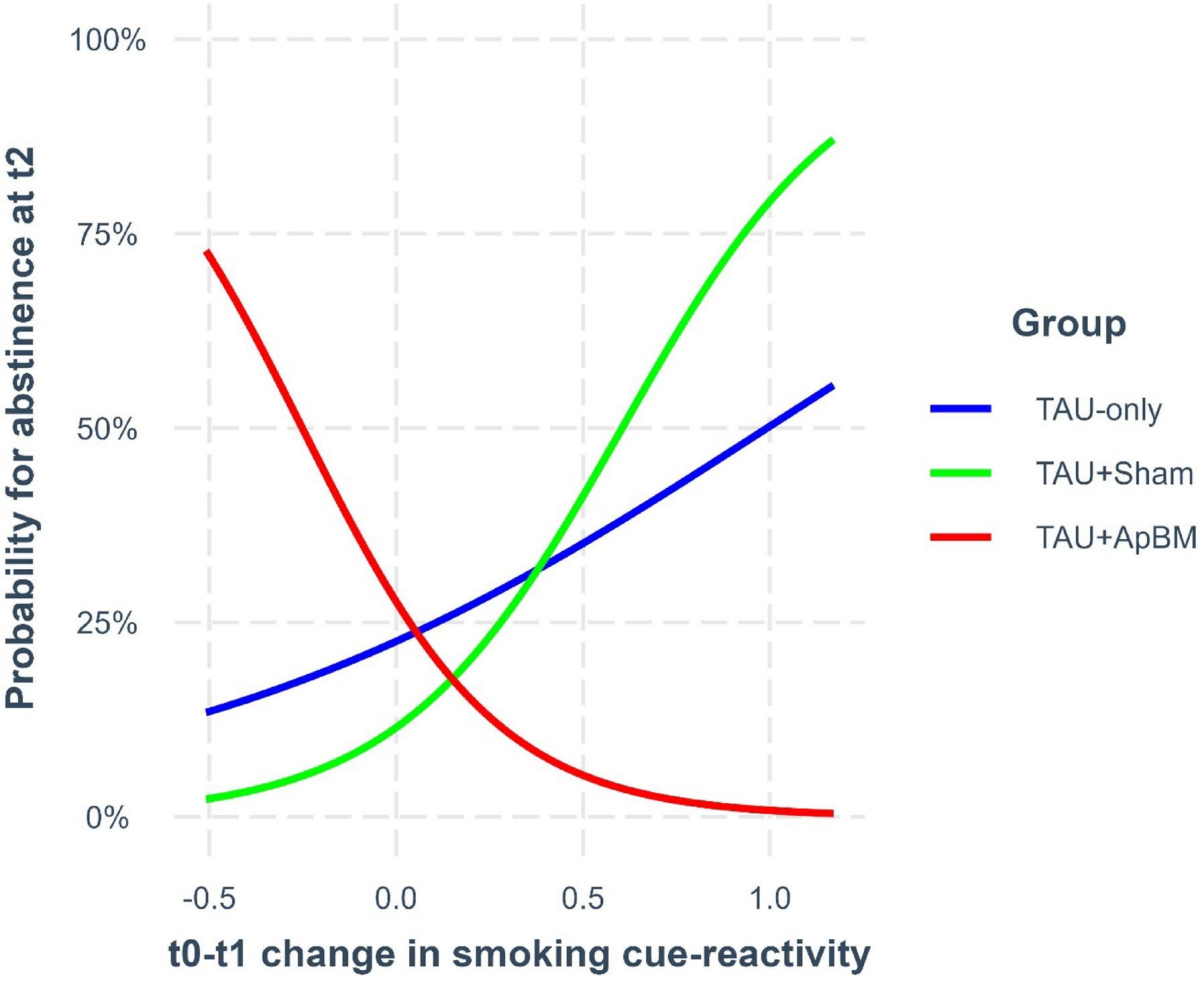
Interaction of group and smoking cue-reactivity changes on long-term abstinence in the right precuneus, Note. Higher positive values in t_0_-t_1_ change in smoking cue-reactivity indicate a greater decrease in smoking cue-reactivity, whereas higher negative values indicate a greater increase. TAU = Treatment-as-usual; ApBM = Approach bias modification; t_0 _= Baseline; t_1 _= Post-intervention; t_2_ = 6-month follow-up.

Regarding long-term abstinence, significant interactions between group and cue-reactivity changes were found in the right precuneus (*p *= .007; see Fig. 4 [interaction plot] and supplementary results Table S1) and right supramarginal gyrus (*p *= .042), both non-significant after FDR-correction. Estimated marginal trends indicated that, in the TAU+ApBM group, increased cue-reactivity in the right precuneus was significantly associated with higher probability of long-term abstinence (logit-scale slope = -3.82, *95%CI*[-7.06 to -0.59]). In contrast, both control groups showed non-significant patterns in the opposite direction, with decreased cue-reactivity marginally linked to higher abstinence probability (TAU+Sham: logit-scale slope = 3.38, *95%CI*[-0.97 to 7.72]; TAU-only: logit-scale slope = 1.24, *95%CI*[-1.13 to 3.61]). These findings suggest that ApBM training may moderate the link between cue-reactivity and long-term abstinence in the right precuneus. Regarding the right supramarginal gyrus, estimated marginal trends did not reach statistical significance in any group (TAU+ApBM: logit-scale slope = -3.56, *95%CI*[-7.28 to 0.16]; TAU+Sham: logit-scale slope = 2.54, *95%CI*[-1.28 to 6.36]; TAU-only: logit-scale slope = 1.61, *95%CI*[-1.83 to 5.05]). Across the whole sample, no significant main effects of cue-reactivity changes on long-term abstinence were observed (*p*s ≥ 0.053).

### Hypothesis 3: Behavioral variables and associations with smoking cue-reactivity changes

At t_0_, participants pushed smoking-related stimuli faster than they pulled them, resulting in a significant negative AAT effect score (avoidance bias; *Z *= -3.50, *p *< .001, *r* = .27; see Table 2 for descriptive statistics). Craving ratings during the smoking cue-reactivity task were significantly higher after smoking-related than neutral blocks (*Z* = 8.01, *p *< .001, *r* = .75). No significant group differences were observed in either behavioral variable at baseline (*p*s ≥ 0.299). Regarding changes from t_0_ to t_1_, both AAT effect scores and craving ratings after smoking-related blocks significantly decreased across groups (AAT effect scores: *p *= .019; craving rating: *p *< .001; see Table 2). This suggests stronger avoidance biases and reduced craving in response to smoking-related stimuli following the interventions. However, reductions did not significantly differ between groups (AAT effect score: *p = *.151; craving ratings: *p = *.091), indicating similar decreases in both AAT effect scores and craving ratings from t_0_ to t_1_ across groups.

Contrary to our hypothesis, no significant group×cue-reactivity change interactions were observed for changes in AAT effect scores or craving ratings in any ROI (*p*s ≥ 0.107; see supplementary results Table S2). Likewise, when averaged across groups, cue-reactivity changes in ROIs were not significantly associated with changes in AAT effect scores or craving ratings (*p*s ≥ 0.096), with one exception: an omnibus test indicated a significant association between cue-reactivity changes in the left anterior cingulate cortex and changes in craving ratings (*p *= .013; non-significant after FDR-correction). Estimated marginal trends showed that, across groups, greater reductions in left anterior cingulate cortex cue-reactivity were associated with greater reductions in craving ratings (slope = 32.9, *95%CI*[12.3 to 53.5]).

In addition to behavioral measures, supplementary analyses were conducted for smoking-related variables as outcomes (cigarettes per day, tobacco dependence severity, CO value). Neither group×cue-reactivity change interactions nor main effects of cue-reactivity changes were significantly associated with changes in smoking-related variables in any ROI (*p*s ≥ 0.073; see supplementary results Table S3). This indicates that changes in neural smoking cue-reactivity were not detectably related to smoking behavior or dependence severity in the present sample.

### Reliability of measures

The reliability of AAT effect and CDS-12 scores (tobacco dependence severity) was estimated as good-to-excellent^53^(≥ 0.64; see supplementary results Table S4). Smoking cue-reactivity measures showed poor^53^ reliability, ranging from -0.36 to 0.28.

## Discussion

This fMRI study investigated the effects of ApBM on neural smoking cue-reactivity in a subsample (*N* = 117) of participants from a randomized-controlled trial, examining the efficacy of ApBM as an add-on to smoking cessation treatment (TAU) in adults with chronic, moderate-to-heavy tobacco dependence. No convincing evidence was found that TAU+ApBM reduced smoking cue-reactivity compared to TAU+Sham and TAU-only. This aligns with clinical outcomes showing no additional benefit of ApBM^25^. Overall, our results contrast with previous findings of reduced alcohol cue-reactivity in reward-related regions after ApBM^19^. Potential explanations for these discrepancies are discussed.

First, chronic smoking may involve neural mechanisms that differ from those underlying alcohol use disorder. ApBM is based on the assumption that substance use disorders are driven by strong approach biases toward drug cues^10^, mediated by heightened activity in subcortical mesolimbic structures^5^. However, we observed *hypo*activation in mesolimbic regions (e.g., thalamus, striatum) in response to smoking-related versus neutral stimuli, challenging this assumption in the context of chronic smoking. Instead, *hyper*activation was found in the left anterior and right middle cingulate cortex, right precuneus, and right supramarginal gyrus—regions associated with response preparation (precuneus, supramarginal gyrus)^54^ and motor coordination and control (anterior and middle cingulate cortex)^55,56^. This suggests that, in individuals with chronic dependence, smoking-related stimuli may elicit automatized, habitual motor responses rather than incentive salience processes^54^. Interventions targeting the inhibition of automatized, habitual actions may prove more effective and merit further investigation.

Second, the smoking-related stimuli used in the ApBM training may have lacked personal relevance, limiting their ability to elicit and modify reward-related neural reactivity. Given the habitual nature of heavy and chronic smoking^57^, incorporating individualized and environmental smoking cues may be crucial to eliciting appetitive responses^58^. Third, unlike C. E. Wiers et al.^19^ who conducted ApBM in a clinical setting with alcohol-abstinent inpatients, the training in our study was delivered at home. Evidence suggests that interventions like ApBM are more effective in institutional settings^59^. It may therefore be warranted to examine the neural effects of smoking ApBM with personalized and contextualized stimuli, and delivered in an institutional setting.

While no significant group effects on smoking cue-reactivity changes emerged, reactivity decreased in the left anterior and right middle cingulate cortex, right precuneus, and right supramarginal gyrus across the whole sample. Accordingly, cue-reactivity reductions within regions involved in response preparation (precuneus, supramarginal gyrus)^54^ and motor coordination and control (anterior and middle cingulate cortex)^55,56^ may be relevant for smoking cessation; however, their specific role remains unclear, due to limited associations with clinical and behavioral outcomes. Nevertheless, three observed associations warrant attention (all non-significant after FDR-correction). First, a greater reduction in anterior cingulate cortex cue-reactivity was associated with stronger craving reduction post-intervention. This finding supports prior evidence on the role of the anterior cingulate cortex in subjective cue-induced craving^60^. Second, a greater decrease in amygdala cue-reactivity was linked to a higher probability of short-term abstinence (post-intervention). This suggests that reduced incentive salience attribution may facilitate short-term smoking cessation^61^. Third, preliminary evidence indicates that ApBM may moderate the relationship between long-term abstinence probability and cue-reactivity in the precuneus. Specifically, among participants who received TAU+ApBM, *increased* precuneus cue-reactivity predicted higher abstinence probability at the 6-month follow-up. In contrast, in the TAU+Sham and TAU-only groups, lower cue-reactivity was descriptively associated with higher abstinence probability, although this pattern did not reach statistical significance. The precuneus is implicated in visuomotor processes, such as integrating visual input with motor planning and preparing automatized motor responses, such as movement guidance towards an object^54,62^. Accordingly, the moderating effect of ApBM may indicate that consistently executing avoidance (i.e., “push”) movements toward smoking-related stimuli strengthened automatized tendencies to disengage from such cues. Over time, this may have facilitated avoidance responses when encountering smoking-related cues in daily life, thereby promoting long-term abstinence. However, this interpretation remains speculative, and the absence of overall ApBM superiority suggests that heightened smoking cue-reactivity in visuomotor areas may only benefit a small subgroup, requiring further research.

### Limitations

Our results should be interpreted against important limitations. First, the time since participants’ last cigarette before the scan session was not assessed for consideration in statistical analyses, as recommended for drug cue-reactivity studies^63^. Consequently, participants’ nicotine satiation status at the time of scanning remains unknown. Variability in nicotine deprivation and associated acute withdrawal symptoms may influence neural responses to smoking cues, particularly within reward- and craving-related circuits, and could therefore have contributed to variability in cue-reactivity across participants^6^. Second, the cue-reactivity paradigm included both trained smoking-related stimuli from the ApBM training and untrained smoking-related stimuli. This ensured consistency between the AAT and cue-reactivity paradigm, facilitating the analysis of behavioral and neural reactivity to the same stimuli. However, since stimuli were randomly presented across smoking-related blocks, it was not possible to analyze training effects or generalization to untrained stimuli. Thus, it remains unclear whether observed neural changes reflect training-specific effects or generalized responses. Third, the split-half reliability of cue-reactivity outcome measures in each ROI was unsatisfactory^53^, potentially leading to spurious findings or obscure effects^64,65^. Thus, findings should be interpreted with caution, and replication is needed to draw firm conclusions. Overall, low reliability appears to be a common issue in drug cue-reactivity analyses, underscoring the need for systematic improvements in the reliability of outcome measures^66,67^.

## Conclusion

This study is the first to examine the effects of ApBM on neural smoking cue-reactivity. Unlike findings in the context of alcohol, our results do not provide convincing evidence that ApBM reduces cue-reactivity in reward-related brain regions. Future research should explore alternative intervention targets (e.g., automatized, habitual motor responses toward smoking-related stimuli) and investigate the efficacy and neural mechanisms of training procedures aimed at these processes.

## Supplementary Information

Below is the link to the electronic supplementary material.

Supplementary Material 1

Supplementary Material 2

Supplementary Material 3

Supplementary Material 4

## Data Availability

Data and analysis code are available in the open science framework (OSF): https://osf.io/zcrbt.
