# Supplementary material for: The effects of approach bias modification on smoking cue-reactivity in individuals who smoke: A randomized controlled fMRI study
Source: Sci Rep. 2026 Mar 28;16:10519. doi: 10.1038/s41598-026-45748-y (PMC13035900; doi:10.1038/s41598-026-45748-y)
Supplement: Supplementary file 1 — Supplementary Material 1 [file 41598_2026_45748_MOESM1_ESM.pdf]

The Effects of Approach Bias Modification on Smoking Cue-Reactivity in Individuals who Smoke: A Randomized Controlled fMRI Study

Supplementary Material

Table of Content

Supplementary Methods **2**
Smoking Cessation Intervention (TAU) 2
Approach-Avoidance Task 2
Task Description 2
Preprocessing 3
fMRI Data Acquisition 3
Supplementary Results **4**
Figure S1 4
Table S1 5
Table S2 6
Table S3 7
Table S4 8
References **9**

Supplementary Methods

Smoking Cessation Intervention (TAU)

The first section of the smoke-free course aims to enhance motivation by addressing ambivalence and increasing self-awareness of smoking behavior through psychoeducation and self-monitoring. The second section focuses on strengthening motivation by reducing anxiety, strengthening confidence in quitting abilities, and initiating the quit attempt. The third section prepares participants for the first 24 hours post-cessation by providing psychoeducation on craving, high-risk situations, and relapse prevention, along with coping strategies (e.g., emergency kit). The final section supports the development of a “non-smoker identity” and reinforces confidence[1]. Participants are offered a follow-up telephone counseling session one week after the course (e.g., to discuss negative side effects such as depressed mood). Smoke-free courses were conducted at the Department of Psychology, LMU Munich, and the outpatient treatment center for tobacco dependence at the LMU University Hospital Munich.

Approach-Avoidance Task

Task Description

The AAT included 80 stimuli (40 smoking-related, 40 positive) sourced from previous studies[2,3] and online platforms. Stimuli were presented in a pseudo-randomized order, ensuring no more than three consecutive pictures from the same category. The task comprised two blocks of 80 trials, with each stimulus appearing once per block. Participants followed a content-relevant feature task instruction, pushing or pulling the joystick based on the picture content (e.g., pushing smoking-related pictures and pulling positive ones, or vice versa). Instruction order (incongruent: push smoking-related stimuli first; congruent: pull smoking-related stimuli first) was counterbalanced across participants and switched after the first block. Response direction was linked to a “zoom” function, where pushing reduced and pulling enlarged the picture. Each trial began with a black screen. Participants pressed the “fire” button while keeping the joystick centered to initiate stimulus presentation, then

responded by pushing or pulling the joystick. Pictures remained on-screen until the correct movement was executed. Before each block, participants completed six practice trials. The AAT was programmed in Visual Basic.

Preprocessing

AAT preprocessing followed the preregistered procedure (see Wittekind et al.[4]). Only correctly executed trials (i.e., without any initial joystick movements in the incorrect direction) were considered. Reaction times below 200 ms or exceeding 2.5 *SD* above the group mean were excluded. Following previous AAT studies (e.g., Wiers et al.[5]), participants with more than 35% missing trials were excluded from further analysis (t_0: $n = 1$; t_1: $n = 1$).

fMRI Data Acquisition

Data were collected using a 3T Siemens Magnetom Prisma with a 32-channel head coil (Siemens AG, Erlangen, Germany). Functional sequences included 320 volumes obtained via a T2*-weighted echo-planar imaging (EPI) sequence (48 slices per volume, ascending interleaved order, multiband factor = 4, voxel size = 3 mm^3 isotropic, repetition time = 2000 ms, echo time = 30 ms, flip angle = 45°, field of view = 210 mm). The first five functional volumes were discarded to mitigate T1 saturation artifacts. High-resolution anatomical images comprised 160 T1-weighted slices acquired with a magnetization-prepared rapid acquisition gradient-echo sequence (voxel size = 1 mm^3 isotropic, repetition time = 2300 ms, echo time = 2.98 ms, flip angle = 9°, field of view = 256 mm).

Supplementary Results

Figure S1

Boxplots of Smoking Cue-Reactivity Changes from t_0 to t_1 in each ROI per Group

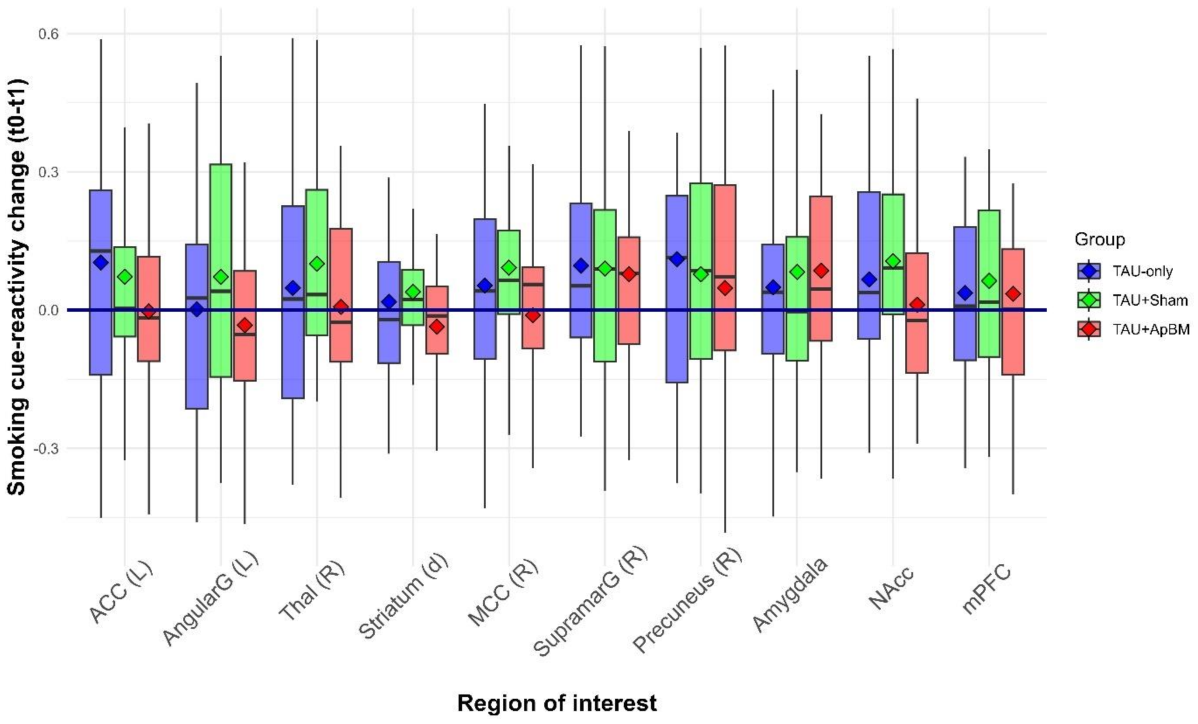

Note. Within boxplots, – represents the median, while ◆ denotes the mean. Higher positive values indicate a greater decrease in smoking cue-reactivity from t_0 to t_1, whereas higher negative values indicate a greater increase. R=right; L=left; ACC=anterior cingulate cortex; AngularG=Angular gyrus; Thal=Thalamus; d=dorsal; MCC=middle cingulate cortex; SupramarG=Supramarginal gyrus; NAcc=Nucleus accumbens; mPFC=Medial prefrontal cortex; TAU=Treatment-as-usual; ApBM=Approach bias modification.

Table S1

Results of the ANOVAs (Omnibus Tests) for the Effects of Time and Time × Group on Abstinence Probability

Region of interest	Effect	(df_1,df_2)	Abstinence at t_1		Abstinence at t_2	
			F	p	F	p
Left ACC	t_0-t_1 change	1,92	3.07	.083	2.71	.103
	t_0-t_1 change×group	2,92	0.97	.384	1.15	.322
Left angular gyrus	t_0-t_1 change	1,92	0.54	.465	1.47	.228
	t_0-t_1 change×group	2,92	0.30	.740	0.24	.788
Right thalamus	t_0-t_1 change	1,92	0.73	.395	1.60	.209
	t_0-t_1 change×group	2,92	0.42	.659	0.88	.417
Dorsal striatum	t_0-t_1 change	1,92	3.70	.058	1.07	.304
	t_0-t_1 change×group	2,92	0.60	.549	0.69	.502
Right MCC	t_0-t_1 change	1,92	1.01	.318	1.29	.258
	t_0-t_1 change×group	2,92	0.26	.769	1.05	.354
Right supramarginal gyrus	t_0-t_1 change	1,92	0.81	.370	0.79	.377
	t_0-t_1 change×group	2,92	0.53	.592	3.27	**.042**[1]
Right precuneus	t_0-t_1 change	1,92	0.13	.718	1.04	.309
	t_0-t_1 change×group	2,92	0.58	.561	5.25	**.007**[1]
Amygdala	t_0-t_1 change	1,92	4.28	**.041**[1]	3.03	.085
	t_0-t_1 change×group	2,92	1.50	.230	1.41	.250
Nucleus accumbens	t_0-t_1 change	1,91	0.37	.547	<0.01	.991
	t_0-t_1 change×group	2,91	1.45	.239	0.35	.704
Medial prefrontal cortex	t_0-t_1 change	1,91	2.31	.132	3.83	.053
	t_0-t_1 change×group	2,91	3.04	.053	2.44	.092

Note. *df*=Degrees of freedom; t_1=Post-intervention; t_2=Follow-up; ACC=anterior cingulate cortex; MCC=middle cingulate cortex.

[1] non-significant after Benjamini-Hochberg correction.

Table S2

Results of the ANOVAs (Omnibus Tests) for the Effects of Time and Time × Group on Behavioral Variables

Region of interest	Effect	AAT effect score			Craving ratings		
		(df_1,df_2)	F	p	(df_1,df_2)	F	p
Left ACC	t_0-t_1 change	1,91	0.42	.520	1,92	6.40	**.013**[1]
	t_0-t_1 change×group	2,91	1.76	.178	2,92	0.66	.520
Left angular gyrus	t_0-t_1 change	1,91	2.30	.133	1,92	1.31	.254
	t_0-t_1 change×group	2,91	2.29	.107	2,92	0.08	.924
Right thalamus	t_0-t_1 change	1,91	2.60	.110	1,92	0.92	.339
	t_0-t_1 change×group	2,91	1.47	.235	2,92	0.76	.470
Dorsal striatum	t_0-t_1 change	1,91	1.16	.284	1,92	0.003	.956
	t_0-t_1 change×group	2,91	1.11	.334	2,92	0.13	.875
Right MCC	t_0-t_1 change	1,91	2.03	.158	1,92	2.83	.096
	t_0-t_1 change×group	2,91	2.20	.117	2,92	1.74	.181
Right supramarginal gyrus	t_0-t_1 change	1,91	1.68	.194	1,92	2.00	.160
	t_0-t_1 change×group	2,91	1.29	.281	2,92	1.92	.153
Right precuneus	t_0-t_1 change	1,91	0.20	.654	1,92	0.09	.760
	t_0-t_1 change×group	2,91	0.42	.659	2,92	1.76	.179
Amygdala	t_0-t_1 change	1,91	0.55	.460	1,92	0.20	.652
	t_0-t_1 change×group	2,91	0.38	.685	2,92	1.05	.354
Nucleus accumbens	t_0-t_1 change	1,90	1.86	.177	1,91	1.27	.262
	t_0-t_1 change×group	2,90	1.31	.276	2,91	0.66	.521
Medial prefrontal cortex	t_0-t_1 change	1,90	0.53	.467	1,91	2.05	.156
	t_0-t_1 change×group	2,90	0.54	.587	2,91	0.08	.923

Note. *df*=Degrees of freedom; t_0=Baseline; t_1=Post-intervention; AAT=Approach-avoidance task; ACC=anterior cingulate cortex; MCC=middle cingulate cortex.

[1] non-significant after Benjamini-Hochberg correction.

Table S3

Results of the ANOVAs (Omnibus Tests) for the Effects of Time and Time × Group on Smoking-Related Variables

Region of interest	Effect	Cigarettes per day			Tobacco dependence			CO value		
		(df_1,df_2)	F	p	(df_1,df_2)	F	p	(df_1,df_2)	F	p
Left ACC	t_0-t_1 change	1,91	0.27	.605	1,91	1.74	.190	1,92	0.28	.601
	t_0-t_1 change × group	2,91	1.93	.152	2,91	1.30	.278	2,92	0.50	.611
Left angular gyrus	t_0-t_1 change	1,91	0.02	.887	1,91	1.22	.273	1,92	0.86	.357
	t_0-t_1 change × group	2,91	1.84	.164	2,91	2.54	.085	2,92	0.88	.420
Right thalamus	t_0-t_1 change	1,91	1.51	.222	1,91	0.51	.478	1,92	0.06	.808
	t_0-t_1 change × group	2,91	0.43	.649	2,91	0.86	.426	2,92	0.33	.718
Dorsal striatum	t_0-t_1 change	1,91	0.79	.378	1,91	0.82	.368	1,92	0.19	.662
	t_0-t_1 change × group	2,91	0.24	.785	2,91	0.96	.388	2,92	1.02	.366
Right MCC	t_0-t_1 change	1,91	0.39	.535	1,91	0.93	.338	1,92	0.27	.605
	t_0-t_1 change × group	2,91	0.78	.461	2,91	2.21	.116	2,92	0.22	.799
Right Supramarginal gyrus	t_0-t_1 change	1,91	1.73	.192	1,91	0.02	.901	1,92	0.62	.431
	t_0-t_1 change × group	2,91	0.56	.575	2,91	0.95	.390	2,92	0.16	.852
Right Precuneus	t_0-t_1 change	1,91	3.09	.082	1,91	0.09	.764	1,92	3.03	.085
	t_0-t_1 change × group	2,91	0.70	.497	2,91	0.37	.695	2,92	1.36	.263
Amygdala	t_0-t_1 change	1,91	0.01	.903	1,91	2.94	.090	1,92	0.73	.396
	t_0-t_1 change × group	2,91	0.02	.983	2,91	2.15	.122	2,92	0.74	.478
Nucleus accumbens	t_0-t_1 change	1,90	0.67	.414	1,90	<0.01	.999	1,91	1.55	.217
	t_0-t_1 change × group	2,90	0.55	.580	2,90	0.05	.954	2,91	0.59	.558
Medial prefrontal cortex	t_0-t_1 change	1,90	0.39	.532	1,90	2.46	.120	1,91	1.12	.293
	t_0-t_1 change × group	2,90	2.70	.073	2,90	0.62	.541	2,91	1.17	.315

Note. Associations between changes in cue-reactivity and smoking-related variables were assessed using linear mixed-effects models (LMMs), with cue-reactivity change, group, and their interaction as predictors. df = Degrees of freedom; t_0 = Baseline; t_1 = Post-intervention; CO = Carbon monoxide; ACC = anterior cingulate cortex; MCC = middle cingulate cortex.

Table S4

Reliability of Measures

Measure	Time	Reliability
**Questionnaire Measures**		**Cronbach's α [95%CI]**
FTND	t_0	.43 [.26 to .55]
CDS-12	t_0	.76 [.68 to .82]
	t_1	.94 [.92 to .95]
**Behavioral Measures**		**Split-half reliability [95%CI]**
AAT effect score	t_0	.72 [.62 to .83]
	t_1	.64 [.53 to .75]
**Neural Measures**		**Split-half reliability [95%CI]**
Left ACC	t_0	.12 [-.21 to .46]
	t_1	.28 [.07 to .52]
Left angular gyrus	t_0	-.03 [-.37 to .27]
	t_1	.16 [-.05 to .39]
Right thalamus	t_0	-.23 [-.60 to .07]
	t_1	.05 [-.23 to .33]
Dorsal striatum	t_0	-.36 [-.74 to .08]
	t_1	.10 [-.24 to .42]
Right MCC	t_0	-.33 [-.67 to -.02]
	t_1	.06 [-.29 to .42]
Right supramarginal gyrus	t_0	.10 [-.25 to .42]
	t_1	.07 [-.31 to .41]
Right precuneus	t_0	-.14 [-.56 to .21]
	t_1	.17 [-.11 to .48]
Amygdala	t_0	-.20 [-.57 to .18]
	t_1	-.02 [-.39 to .31]
Nucleus accumbens	t_0	-.00007 [-.57 to .51]
	t_1	-.04 [.34 to .34]
Medial prefrontal cortex	t_0	-.01 [-.41 to .36]
	t_1	.08 [-.20 to .34]

Note. FTND = Fagerström Test for Nicotine Dependence; CDS-12 = Cigarette Dependence Scale, 12-item version; AAT = Approach-avoidance task; ACC = anterior cingulate cortex; MCC = middle cingulate cortex; t_0 = Baseline; t_1 = Post-intervention; CI = Confidence interval.

References

1. Wenig, J. R., Erfurt, L., Kröger, C. B. & Nowak, D. Smoking cessation in groups--who benefits in the long term? *Health education research* **28,** 869–878; 10.1093/her/cyt086 (2013).

2. Khazaal, Y., Zullino, D. & Billieux, J. The Geneva Smoking Pictures: development and preliminary validation. *Eur Addict Res* **18,** 103–109; 10.1159/000335083 (2012).

3. Oliver, J. A. & Drobes, D. J. Visual search and attentional bias for smoking cues: the role of familiarity. *Experimental and clinical psychopharmacology* **20,** 489–496; 10.1037/a0029519 (2012).

4. Wittekind, C. E. *et al.* Efficacy of approach bias modification as an add-on to smoking cessation treatment: study protocol for a randomized-controlled double-blind trial. *Trials* **23,** 223; 10.1186/s13063-022-06155-6 (2022).

5. Wiers, R. W., Eberl, C., Rinck, M., Becker, E. S. & Lindenmeyer, J. Retraining automatic action tendencies changes alcoholic patients' approach bias for alcohol and improves treatment outcome. *Psychological science* **22,** 490–497; 10.1177/0956797611400615 (2011).
