# Supplementary material for: The effects of approach bias modification on smoking cue-reactivity in individuals who smoke: A randomized controlled fMRI study
Source: Sci Rep. 2026 Mar 28;16:10519. doi: 10.1038/s41598-026-45748-y (PMC13035900; doi:10.1038/s41598-026-45748-y)
Supplement: Supplementary file 2 — Supplementary Material 2 [file 41598_2026_45748_MOESM2_ESM.pdf]

German Clinical Trials Register

DRKS00019221

The efficacy of Cognitive Bias Modification as an add-on in smoking cessation: A randomized-controlled double-blind study

Organizational Data

DRKS-ID:
DRKS00019221
Recruitment Status:
Recruiting complete, study complete
Date of registration in DRKS:
2019-11-11
Last update in DRKS:
2024-12-19
Registration type:
Prospective

Acronym/abbreviation of the study

CBM

URL of the study

http://www.retrainyourbrain.de

Brief summary in lay language

Tobacco dependence is one of the most common mental disorders in Germany. Although most smokers are aware of the long-term negative health consequences and the majority wants to quit smoking, only a minority achieves long-term abstinence. One possible explanation for this apparent contradiction is provided by studies that have shown that automatic processes play an important role in maintaining tobacco dependence. In recent years, there has been an increasing effort to take these automatic processes into account. The planned randomized controlled, double-blind study will investigate whether the efficacy of an established smoking cessation intervention (smokefree® program) can be increased by combining it with a computer training aiming at changing the more automatic processes. Individuals aged 18 to 70 years who suffer from tobacco dependence

can participate in the study (for further in- and exclusion criteria see below). All participants take part in the smoking cessation intervention, which consists of a block event on a single course day (approx. 7h) and a telephone appointment after approx. one week (maximum 15 minutes). Following the smoking cessation intervention, participants are randomized to one of three conditions on a course-by-course basis: One third of the courses receives a specific computer training in addition to the smoking cessation intervention, one third receives a general computer training in addition to the smoking cessation intervention, and another third does not receive any additional intervention. It is expected that participants who receive the smoking cessation intervention in combination with the specific computer training will have a higher abstinence rate after six months and that participants who relapse will have a significantly lower daily cigarette consumption compared to the other two groups.

Brief summary in scientific language

Tobacco dependence is one of the most common mental disorders in Germany (Jacobi et al., 2014). Although most smokers are aware of the long-term negative health consequences and the majority wants to quit smoking, long-term abstinence is the exception rather than the norm, even after receiving treatment (Mottillo et al., 2009). Evidence-based treatments either aim at a pharmacological substitution or at the modification of the more strategic types of information processing. Automatic processes are currently not taken into account sufficiently. The planned randomized controlled, double-blind study pursues two goals: (1) The study aims to investigate the efficacy of Cognitive Bias Modification as a specific add-on to a well-established smoking cessation intervention in a sample of regular smokers. (2) If Cognitive Bias Modification shows incremental efficacy, possible working mechanisms will be investigated. We hypothesize that the smoking cessation intervention + Cognitive Bias Modification will increase 6-month abstinence rates and will reduce daily cigarette consumption in participants who relapsed.

Health condition or problem studied

ICD-10-GM (translation):
    F17.2
Healthy volunteers:
    No

Interventions, Observational Groups

Arm 1:
    Treatment-as-usual (TAU) + Cognitive Bias Modification (using the Approach-Avoidance Task [AAT]) Treatment-as-usual (Smoke-free® program,): for further information see: https://www.rauchfrei-programm.de/schulungsangebote/beschreibung-fuer-fachleute.html. Add-On (Cognitive Bias Modification [CBM]): During the training, which is based on the Approach-Avoidance Task and adapted for tobacco dependence, participants will be presented smoking-related and positive images in a fully randomized order. Participants will be instructed to either pull the images closer or push the images away with a joystick, depending on the tilt (5% to the left or 5% to the right) of the images. In CBM, the instruction is chosen so that all smoking-related images are pushed away and all positive images are pulled. After having finished the smoking cessation intervention, the CBM training should be conducted daily for one week (240 trials per training session).
Arm 2:
    Treatment-as-usual (TAU) + Sham training (using the Approach-Avoidance Task [AAT]) During the training, which is based on the Approach-Avoidance Task and adapted for tobacco dependence, participants will be presented smoking-related and positive images in a fully randomized order. Participants will be instructed to either pull the images closer or push the images away with a joystick, depending on the tilt (5% to the left or 5% to the right) of the images. In the Sham condition, the instruction is chosen so that 50% of the smoking-related and positive images have to be pulled and 50% of

the smoking-related and positive images have to be pushed. The only difference between the CBM and the Sham training is the contingency (240 trials per training session).

Arm 3:
: Treatment-as-usual (TAU) only Participants who are randomized to the third arm receive the treatment-as-usual (i.e., smoking cessation intervention), but no add-on intervention

Endpoints

Primary outcome:
: Abstinence at the 6-month follow-up, defined by the following criteria (Russell Standard, see West et al., 2005) (7) Sustained period of abstinence (8) Sustained abstinence is defined as smoking not more than five cigarettes from start of the abstinence period and biochemical verification. (9) Biochemische Überprüfung (CO-Test): CO ≤ 9ppm. (10) Intention-to-treat Analyse (11) Berücksichtigung von Teilnehmer/-innen bei der Analyse, die das Studienprotokoll verletzt haben (z.B. Einnahme von Nikotinersatzpräparaten) (12) Die Follow-Up Erhebung sollte verblindet erfolgen (Teilnehmer/-innen und Untersucher/-innen).

Secondary outcome:
: 1. Efficacy Secondary analyses will be carried out to investigate whether the combination of treatment-as-usual + CBM can achieve a greater reduction in daily cigarette consumption, a greater reduction of tobacco dependence (CO measurement, Cigarette Dependence Scale, Brief Questionnaire of Smoking Urges) at the post-assessment and a greater reduction in daily cigarette consumption and tobacco dependence (Cigarette Dependence Scale) at the 6-month follow-up compared to the control groups. Finally, it should be examined whether the interventions have an influence on the subjective impairments caused by physical and psychological symptoms (Brief Symptom Inventory). 2. Working mechanisms Additionally, possible working mechanisms will be investigated. So far, it has not been systematically examined whether effects of CBM (based on the Approach-Avoidance Task) is due to (a) the postulated reduction of implicit approach tendencies to smoke-relevant stimuli (as assessed with the Approach-Avoidance Task) or (b) the change of other processes, for example the devaluation of the stimuli (as assessed with the Implicit Association Task [valence]). Furthermore, it will be investigated whether effects of CBM generalize to another paradigm aiming to assess approach tendencies (as assessed with the Implicit Association Task [approach-avoidance]) generalize and whether reacitvity (psychophysiology EMG, acoustic startle) for smoking-related stimuli changes. Finally, it should be examined whether the automatic processes are affected by treatment-as-usual .

Study Design

Purpose:
: Treatment

Allocation:
: Randomized controlled study

Control:
- Other
- Placebo

Phase:
: III

Study type:
: Interventional

Mechanism of allocation concealment:
: No Entry

Blinding:
: Yes

Assignment:
: Parallel

Sequence generation:
No Entry
Who is blinded:
- Assessor
- Investigator/therapist
- Patient/subject

Recruitment

Recruitment Status:
Recruiting complete, study complete

Reason if recruiting stopped or withdrawn:
No Entry

Recruitment Locations

Recruitment countries:
- Germany

Number of study centers:
Monocenter study
Recruitment location(s):
- University medical center Ludwig-Maximilians-Universität München (Lehrstuhl für Klinische Psychologie und Psychotherapie und Tabakambulanz des Klinikums der Universität, München) München

Recruitment period and number of participants

Planned study start date:
2019-11-13

Actual study start date:
2019-11-13

Planned study completion date:
No Entry

Actual Study Completion Date:
2023-10-14

Target Sample Size:
336

Final Sample Size:
354

Inclusion Criteria

Sex:
All

Minimum Age:
18 Years

Maximum Age:
70 Years

Additional Inclusion Criteria:
i. Age 18-70 years ii. CO ≥ 10 ppm iii. Fagerstrøm Test for Nicotine Dependence ≥ 3 iv. Consumption of at least 10 cigarettes daily within the last 12 months v. No consumption of nicotine replacement therapy (NRT) and no pharmacological smoking cessation therapy 3 months prior to study participation vi. Willingness to abstain from NRT, e-cigarettes and any other smoking cessation intervention during study participation vii. Interest to participate in the smokefree program

Exclusion Criteria

i. Severe psychiatric disorder (e.g., bipolar disorder, psychosis) ii. Severe neurological disorder (e.g., z.B. Morbus Parkinson, multiple sclerosis) iii. Acute suicidality iv. Moderate substance dependence (> criteria fulfilled according to DSM-5; except tobacco) v. Pregnancy or nursing vi. Insufficient knowledge of German language

Addresses

Primary Sponsor

Address:
Ludwig-Maximilians-Universität München
PD Dr. Charlotte Wittekind
Leopoldstr. 13
80802 München
Germany
Telephone:
No Entry
Fax:
No Entry
Contact per E-Mail:
✉Contact per E-Mail
URL:
http://www.lmu.de
Investigator Sponsored/Initiated Trial (IST/IIT):
Yes

Contact for Scientific Queries

Address:
Ludwig-Maximilians-Universität MünchenLehrstuhl für Klinische Psychologie und Psychotherapie
PD Dr. Charlotte Wittekind
Leopoldstr. 13
80802 München
Germany
Telephone:
+49 89 2180 5288
Fax:
No Entry
Contact per E-Mail:
✉Contact per E-Mail
URL:

http://www.psy.lmu.de/klin/

Contact for Public Queries

Address:
Ludwig-Maximilians-Universität MünchenLehrstuhl für Klinische Psychologie und Psychotherapie
PD Dr. Charlotte Wittekind
Leopoldstr. 13
80802 München
Germany
Telephone:
+49 89 2180 5288
Fax:
No Entry
Contact per E-Mail:
✉Contact per E-Mail
URL:
http://www.psy.lmu.de/klin/

Principal Investigator

Address:
Ludwig-Maximilians-Universität MünchenLehrstuhl für Klinische Psychologie und Psychotherapie
PD Dr. Charlotte Wittekind
Leopoldstr. 13
80802 München
Germany
Telephone:
+49 89 2180 5288
Fax:
No Entry
Contact per E-Mail:
✉Contact per E-Mail
URL:
http://www.psy.lmu.de/klin/

Sources of Monetary or Material Support

Government or public funding body, financed by tax revenue (e.g. the German DFG, BMFTR)

Address:
Deutsche Forschungsgemeinschaft
Kennedyallee 40
53175 Bonn
Germany
Telephone:
No Entry
Fax:
No Entry
Contact per E-Mail:
✉Contact per E-Mail
URL:

http://www.dfg.de

Ethics Committee

Address Ethics Committee

Address:
Ethikkommission - Fakultät für Psychologie und Pädagogik
Leopoldstr. 13
80802 München
Germany
Telephone:
+4989218072514
Fax:
No Entry
Contact per E-Mail:
✉Contact per E-Mail
URL:
https://www.fak11.lmu.de/forschung/ethikkommission/index.html

Vote of leading Ethics Committee

Vote of leading Ethics Committee
Date of ethics committee application:
2019-07-05
Ethics committee number:
23_c_2019
Vote of the Ethics Committee:
Approved
Date of the vote:
2019-10-10

Further identification numbers

Other WHO Primary Registry or Data Provider ID:
No Entry
EudraCT Number:
No Entry

UTN (Universal Trial Number):
No Entry
EUDAMED Number:
No Entry

IPD - Individual Participant Data

Do you plan to make participant-related data (IPD) available to other researchers in an anonymized form?:
Yes
IPD Sharing Plan:
The datasets of the current trial will be made publicly available after an embargo period or after publication of the major findings.

Study protocol and other study documents

Study protocols:
Wittekind CE, Takano K, Sckopke P, Winkler MH, Werner GG, Ehring T, Rüther T. Efficacy of approach bias modification as an add-on to smoking cessation treatment: study protocol for a randomized-controlled double-blind trial. Trials. 2022 Mar 21;23(1):223. doi: 10.1186/s13063-022-06155-6. PMID: 35313949; PMCID: PMC8935694.

Study abstract:
No Entry

Other study documents:
No Entry

Background literature:
No Entry

Related DRKS studies:
No Entry

Publication of study results

Planned publication:
No Entry

Publications/study results:
No Entry

Date of the first journal publication of results:
No Entry

DRKS entry published for the first time with results:
No Entry

Basic reporting

Basic Reporting / Results tables:
No Entry

Brief summary of results:
No Entry

The Federal Institute for Drugs and Medical Devices (Bundesinstitut für Arzneimittel und Medizinprodukte, BfArM) is an independent higher federal authority within the portfolio of the Federal Ministry of Health.
© BfArM 2026

- Imprint
- Data Privacy Declaration
- Terms of Use
