# Supplementary material for: The effects of approach bias modification on smoking cue-reactivity in individuals who smoke: A randomized controlled fMRI study
Source: Sci Rep. 2026 Mar 28;16:10519. doi: 10.1038/s41598-026-45748-y (PMC13035900; doi:10.1038/s41598-026-45748-y)
Supplement: Supplementary file 3 — Supplementary Material 3 [file 41598_2026_45748_MOESM3_ESM.pdf]

Wittekind *et al. Trials* (2022) 23:223
https://doi.org/10.1186/s13063-022-06155-6

Trials

STUDY PROTOCOL Open Access

Efficacy of approach bias modification as an add-on to smoking cessation treatment: study protocol for a randomized-controlled double-blind trial

Charlotte E. Wittekind[1*], Keisuke Takano[1], Philipp Sckopke[1], Markus H. Winkler[2], Gabriela G. Werner[1], Thomas Ehring[1] and Tobias Rüther[3]

Abstract

Background: Although effective treatments for smoking cessation are available, long-term abstinence is the exception rather than the norm. Accordingly, there is a need for novel interventions that potentially improve clinical outcome. Although implicit information processing biases, for example approach biases for smoking-related stimuli, are ascribed a dominant role in the maintenance of tobacco dependence, these biases are hardly targeted in current treatment. Past research has shown that so-called Approach Bias Modification (AppBM) trainings, aiming to modify this bias, lead to improved long-term abstinence in abstinent alcoholic inpatients when delivered as an add-on to treatment-as-usual. Findings on the efficacy of AppBM in smoking have been inconsistent. The present large-scale clinical trial pursues two goals. First, it aims to investigate the efficacy of AppBM as an add-on to treatment-as-usual in a representative sample of adult smokers. Second, possible mechanisms of change are investigated.

Methods: The study is a randomized-controlled, double-blind, parallel-group superiority trial. We aim at a final sample of at least 336 adult smokers. Participants are allocated with a 1:1:1 allocation ratio to one of the following conditions: (1) treatment-as-usual + AppBM, (2) treatment-as-usual + Sham, (3) treatment-as-usual only. During the add-on training, participants are presented smoking-related and positive pictures and are instructed to respond by either pushing or pulling a joystick, depending on the tilt of the pictures (5° to the left/right). During AppBM, all smoking-related pictures are tilted in the direction that is associated with pushing, thereby aiming to train an avoidance bias for smoking. All positive pictures are tilted in the direction associated with pulling. During Sham, the contingency is 50/50. Participants are assessed before and after the intervention and at a 6-month follow-up. The primary outcome is prolonged abstinence, and secondary outcomes include smoking-related variables and psychological distress. Additionally, the motivational significance of smoking-related stimuli (i.e., approach bias, valence) is assessed with different experimental tasks (Approach-Avoidance Task; Single Target Implicit Association Test) and psychophysiological measures.

* Correspondence: charlotte.wittekind@psy.lmu.de
[1]Department of Psychology, LMU Munich, Leopoldstraße 13, 80802 Munich, Germany
Full list of author information is available at the end of the article

© The Author(s). 2022 **Open Access** This article is licensed under a Creative Commons Attribution 4.0 International License, which permits use, sharing, adaptation, distribution and reproduction in any medium or format, as long as you give appropriate credit to the original author(s) and the source, provide a link to the Creative Commons licence, and indicate if changes were made. The images or other third party material in this article are included in the article's Creative Commons licence, unless indicated otherwise in a credit line to the material. If material is not included in the article's Creative Commons licence and your intended use is not permitted by statutory regulation or exceeds the permitted use, you will need to obtain permission directly from the copyright holder. To view a copy of this licence, visit http://creativecommons.org/licenses/by/4.0/. The Creative Commons Public Domain Dedication waiver (http://creativecommons.org/publicdomain/zero/1.0/) applies to the data made available in this article, unless otherwise stated in a credit line to the data.

Discussion: This is the first large-scale clinical trial investigating the efficacy of AppBM as an add-on in smokers including a TAU only condition. Additionally, it is the first study to systematically investigate potential mechanisms mediating the effects of treatment on clinical outcome.

Trial registration: German Clinical Trials Register, DRKS00019221, 11/11/2019

Keywords: Smoking, Smoking Cessation, Cognitive Bias Modification, Approach Bias Modification, Approach-Avoidance Task, Electromyography, Acoustic Startle Reflex, Add-On, Randomized-Controlled Trial

Background

Chronic cigarette smoking is associated with severe negative health consequences and remains one of the major preventable causes of premature death [1, 2]. The negative health consequences and the risk of premature death can significantly be reduced if smoking is stopped [3, 4]. According to (inter-)national guidelines, cognitive behavioral therapy, pharmacotherapy, nicotine replacement therapy (NRT), and their combination (CBT + NRT/pharmacotherapy) represent first-line treatments [5–7]. However, even these evidence-based treatments do not achieve satisfactory abstinence rates as long-term effects of CBT and other smoking cessation interventions are only modest [7–9]. Consequently, there is a need for new additional smoking cessation strategies to improve long-term outcome.

One starting point to improve abstinence is to take characteristics of substance-related information processing into account. Dual-process models of information processing [10–12] describe substance use disorders in terms of an imbalance between two interacting systems: an impulsive, associative system, and a reflective, propositional system. The impulsive system operates relatively automatic and is easily activated by stimuli signaling immediate reward or punishment. The reflective system is more resource dependent and comprises cognitive control and decision making processes, which allows to consider long-term outcomes. Proponents of dual-process models suggest that substance use behavior may be characterized by strong, impulsive responses to drug-associated stimuli, which are insufficiently controlled by reflective processes [11, 13]. Although heightened impulsive responses have previously been suggested [13] to be the result of several prominent forms of drug-related learning [14–16], incentive motivational accounts have received increased interest. For instance, Robinson and Berridge highlight the strong motivational effects of drug-related stimuli ("wanting") and hypothesize that the attribution of sensitized incentive salience to drug-related stimuli endows them with the capacity to capture attention and motivate approach [14]. According to dual-process models, enhanced impulsive processing of drug-related stimuli might be "insufficiently" counteracted by weak or weakened reflective processes, e.g., related to cognitive control [13]. Accordingly, deficient reflexive processes have been discussed as a premorbid condition increasing the risk for substance use problems and/or as a result of continuing drug intake [12, 17]. In line with the theoretical assumptions, it has been shown that smoking is related to attentional [18, 19] and behavioral approach biases for smoking-related stimuli [20, 21]. Approach biases are frequently assessed by means of a joystick Approach-Avoidance Task (AAT, [22]). In order to assess behavioral tendencies in smoking, smoking-related and smoking-unrelated stimuli have to be pushed (avoided) and pulled (approached) by means of a joystick. Moreover, the motivational effects of drug-related stimuli have been investigated with psychophysiological measures. For example, using facial electromyography (EMG), it has been found that in smokers, smoking cue exposure attenuates the acoustic startle response (ASR, [23, 24]) and increases activity of the zygomatic muscle and decreases activity of the corrugator muscle, indicative of enhanced positive affect and reduced negative affect [25].

Approach bias modification: rationale and clinical effects

If behavioral approach biases for drug-related stimuli are an important maintenance factor of substance use disorders, their reduction by means of Approach Bias Modification (AppBM) should improve clinical outcome. During AppBM, contingencies in the AAT are modified so that drug-related stimuli are consistently associated with pushing (i.e., avoidance) and drug-unrelated stimuli with pulling (i.e., approach) while the control (Sham) training includes a contingency of 50/50. Sham training has served as the "standard" control training as it includes the same amount of exposure to drug-related stimuli, but should not change behavioral biases. Several studies in patients with alcohol use disorders have shown that the combination of treatment-as-usual (TAU [12-weeks of CBT-based, abstinence-orientated inpatient treatment]) and AppBM improved long-term abstinence in abstinent inpatients [26–28]. In addition, delivering AppBM during detoxification has been shown to improve abstinence [29, 30]. In smoking, evidence for the efficacy of AppBM is mixed. While positive effects of AppBM on daily cigarette consumption [31, 32] and short-term abstinence [33] have been found, studies

including longer-term abstinence (i.e., > 3 months) as the outcome have mostly yielded null findings [34, 35]. To the best of our knowledge, there is only one published study that has investigated the efficacy of AppBM as an add-on to TAU on abstinence in adult participants [34]. Adult smokers received three sessions of a CBT-based smoking cessation intervention and additionally either six sessions of AppBM (*n* = 54) or Sham training (*n* = 51); however, TAU + AppBM was not superior to TAU + Sham at the 6-month follow-up on any clinical outcome. Given that two studies did not find beneficial effects of add-on AppBM on abstinence, one might question whether it is worthwhile to further investigate its efficacy (see [36] for a critical discussion). However, results of an individual patient data meta-analysis suggest that Sham training becomes more effective in reducing relapse rates if the trial number increases [37]. The authors hypothesize that the continuous exposure to drug-related stimuli during many trials of Sham training results in decreased reactivity for drug-related stimuli. As two studies with null findings [34, 35] had a higher number of trials than studies with positive outcomes [27, 29, 32, 33], it might be conceivable that both AppBM and Sham training exerted positive effects on abstinence rates. However, as no TAU only condition was included, this question cannot be answered. As the individual patient data meta-analysis also revealed small effects on cognitive bias change (collapsed across different forms of training) and small, but unreliable effects on relapse [37], it appears to be highly important to further investigate whether AppBM and/or Sham training can increase long-term abstinence in smoking cessation in a large-scale clinical trial.

Working mechanisms

Cognitive bias modification (CBM) procedures have originally been developed to test the causal relation between cognitive biases and emotional vulnerability [38]. The seminal studies by MacLeod et al. have stimulated a wealth of CBM studies investigating whether the modification of information processing biases exerts positive effects on a wide range of psychological problems [37, 39, 40]. The central tenet of CBM is that effects of training procedures on outcome are mediated by a bias change. Although formal tests of mediation are scarce, it has been shown that if the underlying bias is successfully modified, positive effects on emotional vulnerability emerge [41, 42]. AppBM, which represents a subcategory of CBM procedures, should result in a reduction of approach biases for drug-related stimuli which, in turn, should exert positive effects on substance consumption and abstinence [40].

However, this assumption has not been supported by studies on AppBM in smoking [32, 34]. More specifically, although Machulska et al. [32] found a significant direct effect for the association between training condition (AppBM vs. Sham) and nicotine consumption, the indirect effect was not significant indicating that positive effects of training condition on outcome were not mediated by a change in approach bias. In the Wittekind et al. study [34], training condition predicted a change in the smoking approach bias; however, the AAT bias change was not related to clinical outcome. A similar pattern emerged in alcohol AppBM research: Training condition predicted changes in alcohol approach biases, but this bias change was not related to outcome [27]. However, in a replication study with a larger sample, support for a mediation (indirect effect) was found [28], as training condition was a predictor of change in alcohol approach biases, and this change, in turn, predicted treatment outcome. However again, in a recent large-scale study investigating the effectiveness of alcohol AppBM, alcohol attention retraining, and the combination of AppBM + attention retraining, no evidence for a specific mediation could be revealed [26]. Taken together, previous findings cast doubt on the rationale that positive effects of AppBM on outcome are mediated by a change in approach biases. Therefore, to understand *why* AppBM has been effective in improving clinical outcome, more research investigating *how* AppBM affects the processing of drug-related stimuli is needed.

For example, Wiers et al. [27, 43] showed that AppBM led to changes in the accessibility of associations between alcohol-related stimuli and the semantic concepts of approach vs. avoidance as indicated by an Implicit Association Test (IAT). In this regard, it might be important that another study, which re-analyzed data of Wiers et al. [27], provided preliminary evidence that alcohol-avoidance associations (as assessed with the IAT) predicted lower relapse [44]. It is also conceivable that effects of AppBM result from a devaluation of smoking-related stimuli. More specifically, the Behavior Stimulus Interaction (BSI) theory proposes that if approach behavior for an attractive stimulus is inhibited the stimulus is devalued (i.e., gets more negative, [45]). This assumption has been empirically supported in a series of studies showing that if participants are instructed to withhold a response to attractive stimuli, for example during a no-go trial in the Go/No-Go Task, a devaluation of these no-go stimuli occurs [45, 46]. Consequently, pushing substance-related stimuli away might result in a similar effect, and there is preliminary evidence showing that AppBM increased negative attitudes towards smoking as assessed with an attitudinal IAT in subgroups of participants [47]. Furthermore, psychophysiological measures are included to investigate whether AppBM affects the motivational significance of smoking-related stimuli.

Trial objectives

The aims of the 1:1:1 parallel-group superiority randomized-controlled, double-blind trial are twofold: First, it aims to investigate the efficacy of AppBM as an add-on to a CBT-based smoking cessation intervention. In our prior study [34], in which TAU + AppBM was not superior to TAU + Sham on any clinical outcome, three training sessions were conducted before and three training sessions after the quit attempt, which might have compromised the efficacy of AppBM. Alternatively, the lack of between-group differences might be explained by the high number of training trials so that Sham training might have exerted positive effects on abstinence [37]. As a consequence and in line with studies in abstinent alcoholic inpatients [26–28], all trainings sessions are conducted *after* the quit attempt in the present study. Additionally, to test whether both AppBM and Sham training affect clinical outcome, a control condition without additional training (TAU only) is included. The primary outcome is abstinence rates at the 6-month follow-up. We expect that TAU + AppBM results in significantly larger abstinence rates at the 6-month follow-up compared to TAU + Sham and TAU only.

Second, the trial aims to increase our understanding of the working mechanisms of AppBM by studying its effects on different components of information processing. Given the traditional CBM rationale, effects of AppBM on abstinence and secondary clinical outcomes should be mediated by a reduction of approach biases for smoking-related stimuli; however, this assumption has not consistently been supported by empirical evidence. In the current study, the motivational significance of smoking-related stimuli is assessed with different experimental paradigms (AAT, Single Target IAT [ST-IAT]) and with psychophysiological measures (EMG). Based on the rationale of CBM, the main hypothesis would be that effects of AppBM on abstinence are mediated by a change in smoking approach biases. Alternative hypotheses are that effects of TAU + AppBM on abstinence are mediated by (a) an increase of tobacco-avoidance associations (assessed with an approach-avoid ST-IAT), (b) a devaluation of smoking-related stimuli (assessed with a valence ST-IAT), or (c) a reduction of the motivational significance of smoking-related stimuli (assessed with EMG). The design of the present study can also answer whether both trainings exert positive effects on relapse, but work through different mechanisms.

Method

Design and setting

This study is designed as a randomized, controlled, double-blind, single-center superiority trial with three parallel groups. The trial is conducted in Germany at the Department of Psychology at the LMU Munich and at the outpatient treatment center for tobacco dependence of the university hospital Munich. The primary endpoint is prolonged abstinence at the 6-month follow-up. Randomization is performed by an external study center as a block randomization with a 1:1:1 allocation ratio. Participants are randomly allocated to one of three groups: (1) TAU + AppBM, (2) TAU + Sham, and (3) TAU only. The proposed flow of participants is shown in Fig. 1. The study protocol was written in compliance with the SPIRIT guidelines [48], see Appendix 1 for the SPIRIT checklist.

Participants

To be included in the study, the following inclusion criteria have to be met: (1) age 18–70 years, (2) a total score of ≥ 3 in the Fagerstrøm Test for Nicotine Dependence (FTND [49]), (3) a value of ≥ 10 ppm carbon monoxide (CO) in exhaled air, (4) smoking ≥ 10 cigarettes daily within the past 12 months, (5) no consumption of nicotine replacement therapy (NRT) and no pharmacological smoking cessation therapy three months prior to study participation, (6) willingness to abstain from NRT, e-cigarettes, and any other smoking cessation interventions during study participation, and (7) interest to participate in the smoke-free® program. The following exclusion criteria are applied: (1) severe psychiatric (e.g., bipolar disorder, psychosis) or neurological disorder (e.g., multiple sclerosis, Morbus Parkinson); (2) acute suicidality; (3) moderate substance dependence within the last 12 months (i.e., ≥ 4 criteria according to DSM-5 [50], except tobacco dependence; (4) pregnancy or breast feeding; and (5) insufficient knowledge of German language.

Sample size

We based our power calculation on the findings of a similar intervention study reporting a significant training effect on daily cigarette consumption [32][1]. Our planned analysis is multilevel modeling with Time (pre-intervention, post-intervention, follow-up) as the within-person factor and with Group (TAU + AppBM, TAU + Sham, TAU only) as the between-person factor. As Machulska et al. [32] did not include a TAU only group, we assumed that this group has the same mean and standard deviation as the Sham control group. We fitted the multilevel model on Machulska et al.'s data, which was used as a prior to perform power calculations with

[1] At the moment of planning the study, there was only one published study [32] in which a short CBT intervention was combined with AppBM vs. Sham training in a sample of adult smokers. As the study used number of daily cigarettes as the primary outcome, but not abstinence rates, the power calculation was based on the number of daily cigarettes.

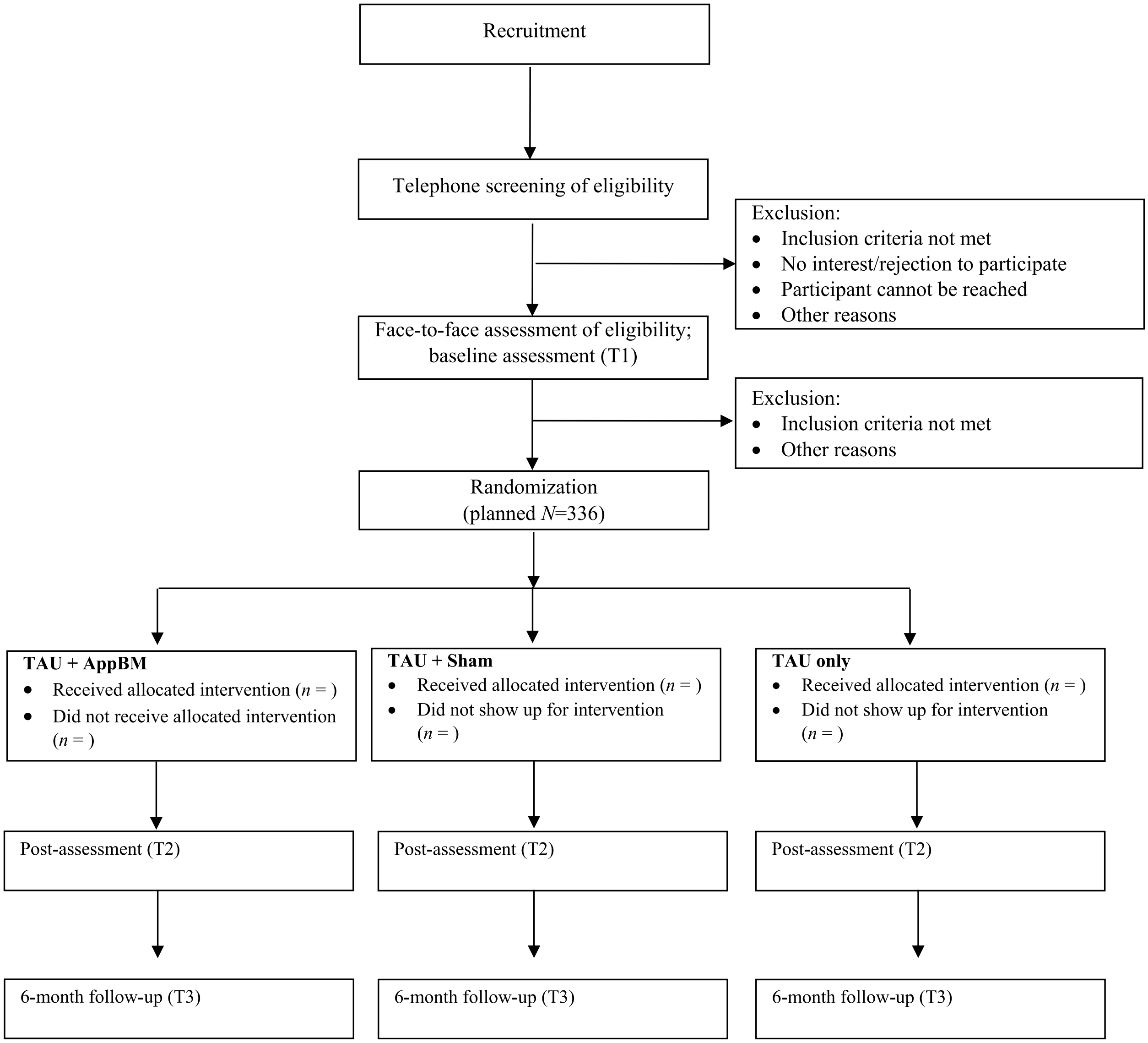

Fig. 1 Proposed flow of participants. AppBM = Approach Bias Modification

Monte Carlo Simulations (using the R package simr, [51]). The expected effect size is d = 1.07 (i.e., d_GMA-change, [52]) for a Time × Group interaction. To identify the minimum sample size required to detect the expected effect with α = .05, we simulated the power while increasing the sample size from n = 3 to 600. The power curve indicated a power of .78 at N = 268, which we regarded as the minimum sample size required. Assuming a drop-out rate of 20%, a total sample size of 336 is needed (i.e., 112 participants per condition). As the primary outcome of the study is prolonged abstinence at the 6-month follow-up, a minimum detectable effect sizes (MDES) analysis was conducted to examine which effect size can be obtained with a sample size of N = 268, α = .05, and 1-beta = .80. The analysis resulted in a small effect of ω = .19, which is comparable to the effect sizes found in previous studies [27, 28].

Recruitment and procedure

On average, one to two smoke-free interventions are offered per month. For each intervention, up to 14 participants are recruited via advertisements in social media (e.g., Facebook), online marketing tools (e.g., Google AdWords), university websites, posters and flyers distributed at, for example, the campus and medical practices. If these strategies do not suffice, further strategies are used. The study website contains detailed information about the study (e.g., aims, procedure, randomization, contact information), flyers, posters, a short description of the study, and contact details. Interested parties are

asked to get in touch with the study team and are first contacted by phone. During the telephone screening, participants are informed in more detail about the aims and the procedure of the study and about in- and exclusion criteria. If participants seem eligible, an appointment for the baseline assessment is made. All participants recruited for one course are assessed during the 2-week period before the 1-day smoke-free intervention takes place. At the beginning of the baseline assessment, in- and exclusion criteria are checked with an interview assessing sociodemographic and smoking-related information. Additionally, the section on substance dependence of the Mini International Neuropsychiatric Interview (MINI [53]) is conducted to screen for any substance dependence, the FTND is administered, and CO is measured. Only if all inclusion criteria are met, the baseline assessment is continued (see below for measures). All participants recruited for a specific course who fulfill inclusion criteria receive the 1-day smoke-free intervention; the entire course is randomized to one of the three arms (TAU + AppBM, TAU + Sham, TAU only). Participants are informed whether they receive an additional training or not *after* completing the smoke-free intervention in order to prevent different expectancy effects across groups. Participants who are randomized to one of the trainings (AppBm, Sham) in addition to TAU conduct the first training session subsequent to the smoke-free intervention and are asked to perform the training daily for the following 7 days from their home computer. One week after the smoke-free intervention, participants receive an individual telephone consultation (about 15 min). Upon completion of the telephone consultation, the post-assessment (see Table 1) takes place (approx. 2–3 weeks after baseline). Participants are re-assessed 6 months after the quit attempt (follow-up). Following an intention-to-treat (ITT) approach, all randomized participants are asked to complete the assessments (i.e., this also includes those participants who do not adhere to or violate the treatment protocol, e.g., no attendance of smoke-free intervention; usage of NRT during study participation). All assessments take place face-to-face at the Department of Psychology at the LMU Munich.

Corona pandemic specifics

Due to the corona pandemic and closing of university buildings, a subgroup of participants is asked to complete the follow-up assessment online. The smoking cessation intervention has to be administered online for several courses as group meetings are temporarily not permitted. Also, during the restrictions, participants were not allowed to remove their masks. This means the collection of EMG data as part of the Passive Picture Viewing Task was not possible; therefore, the task was not administered during the strict restrictions. The original study procedures will be reinstated as soon as the pandemic-related restrictions will be lifted.

Measures

Sociodemographic, smoking-related, and psychopathological information

Relevant sociodemographic (e.g., age, sex, education, handedness) and smoking-related information (e.g., smoking duration, cigarettes smoked per day [CPD], number of prior quit attempts) are collected using a short interview. During the interview, information on medication, suicidality, specific health-related information (e.g., allergies; ear problems [e.g., tinnitus], neurological, and psychiatric disorder), information regarding the female menstrual cycle, and in-/exclusion criteria are assessed. Substance dependence is inquired with the respective sub-section of the MINI.

To screen for mental disorder (depression, generalized anxiety disorder, panic disorder with and without agoraphobia, social phobia, specific phobia, obsessive compulsive disorder, posttraumatic stress disorder, and alcohol abuse/dependence), the Web Screening Questionnaire for Common Mental Disorders (WSQ [54]) is administered. To assess subjective constrains due to physical and psychological symptoms, participants are ask to complete the Brief Symptom Inventory (BSI [55, 56]).

Assessments and outcome measures

The main outcome measures are assessed pre-intervention, post-intervention, and at the 6-month follow-up; an overview of all measures and their administration is provided in Table 1.

Assessments of sample characteristics and training evaluation The 6-item FTND [49] is administered to ensure that participants are eligible for the study. A recent meta-analysis has shown that the FTND is a reliable instrument [57]. As impulsivity has been shown to be associated with drug-related approach biases [58], the Barratt Impulsiveness Scale-15 (BIS-15, German version: [59, 60]) is used in order to compare groups as to their impulsivity. It consists of 15 items and three subscales (non-planning impulsivity, motor impulsivity, attentional impulsivity). To account for individual differences regarding inhibition (i.e., suppression of pre-potent responses, [61]), the color Stroop task is administered pre-intervention. At the post-intervention assessment, participants are asked to answer several questions pertaining to the interventions and abstinence (see Supplementary Material).

Primary outcome The primary outcome of the study is prolonged abstinence at the 6-month follow-up. Long-

Table 1 Overview of all measures

Timepoint	S	T1 (weeks 1–2)	Week 3	T2 (weeks 4–5)	T3 (6 months)
**Enrollment**					
Eligibility screen	X				
Informed consent		X			
Allocation		X			
**Interventions**					
TAU + AppBM			↔		
TAU + Sham			↔		
TAU only			X		
**Assessments**					
Baseline Assessment					
Demographics		X			
Smoking-related information		X			
Substance dependence (MINI)		X			
FTND		X			
Suicidality		X			
BIS-15		X			
WSQ		X			
Stroop		X			
Primary Outcome					
Abstinence (6-month FU)				X	X
Secondary Outcomes					
BSI		X		X	
QSU-brief		X		X	
Smoking behavior		X		X	X
CO		X		X	X
CDS-12		X		X	X
Questionnaire training				X	
Working Mechanisms					
AAT		X		X	X
ST-IAT approach-avoid		X		X	
ST-IAT valence		X		X	
Passive Picture Viewing Task		X		X	

Note. S = screening; T1 = pre-intervention (baseline); T2 = post-intervention; T3 = 6-month follow-up
TAU treatment-as-usual, *AppBM* Approach Bias Modification, *MINI* Mini International Neuropsychiatric Interview, *FTND* Fagerstrøm Test for Nicotine Dependence, *BIS-15* Barratt Impulsiveness Scale-15, *WSQ* Web Screening Questionnaire, *BSI* Brief Symptom Inventory, *QSU-brief* Brief Questionnaire of Smoking Urges, *CO* carbon monoxide, *CDS-12* Cigarette Dependence Scale-12, *AAT* Approach-Avoidance Task, *ST-IAT* Single Target Implicit Association Test

term abstinence is assessed following recommendations by West et al. [62], defined by the Russell Standard (RS) which incorporates six criteria: (1) continuous abstinence of 6 months starting with the quit date, (2) RS abstinence, that is, not having smoked more than five cigarettes (self-report) in combination with a negative biochemical validation (i.e., CO) at follow-up, that is, (3) CO in expired air of ≤ 9 ppm. If no objective verification of smoking status can be obtained, participants will be classified as smoking, (4) all randomized participants will be included for analyses (exception: participant died during study participation; moved to unknown address), (5) protocol violators (e.g., usage of NRT) will also be included for analyses and classified according to their smoking status, (6) blinded follow-up assessment.

Secondary outcomes Smoking-related outcomes are assessed with the following measures.

Tobacco dependence

Participants' tobacco dependence is assessed with the Cigarette Dependence Scale-12 (CDS-12, [63]), a 12-item self-report questionnaire following the diagnostic criteria of the DSM-IV and the ICD-10. Internal consistency can be considered excellent (Cronbach's α = .90); test-retest reliability for the CDS-12 has been estimated at r = .84 [63].

Craving

The 10-item Brief Questionnaire of Smoking Urges (QSU-brief) is administered to measure self-reported craving on two subscales (intention/desire to smoke, relief of negative affect or withdrawal), the total score has shown high internal consistency (Cronbach's α = .87–.97, [64]).

Smoking behavior

Additionally, smoking behavior during the last 7 days is assessed using five items assessing daily cigarette consumption, abstinent days, and usage of other nicotine products (e.g., e-cigarette, nicotine patch, snuff).

Carbon monoxide

The CO concentration in exhaled air is measured with the smokerlyzer® (Micro + Smokerlyzer Bedfont Scientific Ltd., Maidstone, England).

Psychological distress

The BSI [55, 56] is administered to assess psychological distress during the last 7 days on nine subscales with a 5-point Likert scale. The *Global Severity Index* serves as secondary outcome in the present study and has shown excellent internal consistency and test-retest reliability [56].

Experimental tasks

The experimental tasks are conducted in a fixed order (Passive Picture Viewing Task, AAT, ST-IATs, Stroop [pre-intervention only]). The order of instruction in the AAT (pull smoking first, push smoking first), the order of the ST-IATs (valence ST-IAT, approach-avoid ST-IAT), and the order of instructions in the ST-IATs (compatible first, incompatible first) are counterbalanced across participants (see Supplementary Material Table S1).

Approach-Avoidance Task Approach biases for smoking-related stimuli are assessed with the joystick AAT [22] using 80 pictures (40 smoking-related, 40 positive). In our previous study [34], smoking-related pictures were selected from prior studies and depicted rather complex scenes and images (e.g., smoking people in a bar; cup of coffee with a lit cigarette in ashtray). As pictorial stimuli were simple in the alcohol studies [26–28], novel and less complex stimuli were compiled for the present study. Smoking-related stimuli were selected from prior studies [65, 66] as well as the internet and picture agencies (Fotolia; Shutterstock) and depicted packages of cigarettes, burning cigarettes, and an ashtray with cigarettes. Based on one study in smoking [67] which used positive instead of matched control stimuli, 40 positive pictures that are presented during the smoking cessation intervention are used as the reference category (during the intervention, each participant has to pick one out of 40 pictures that she/he associates with a smoke-free future). All selected pictures were rated in an online survey using Unipark® (www.unipark.com) by smokers (for ratings see Supplementary Material Table S2). For the training, 20 smoking-related and 20 positive pictures were randomly selected in order to be able to test generalization. Based on the ratings, it was ensured that trained and untrained pictures were comparable regarding valence, arousal, and craving (for a similar approach see [68]).

Explicit instructions are used such that response direction depends on the content of the pictures. The AAT comprises two blocks with a switch of instruction after the first block, each picture is presented once per block in a pseudo-random order (no more than three pictures of the same category are presented consecutively, see [22]). Order of instruction (pull smoking first vs. push smoking first) is counterbalanced across participants. In order to improve the impression of approach versus avoidance, response direction is linked to a zoom-function such that picture size decreases when the joystick is pushed and increased when the joystick is pulled. In each block, six practice trials precede 80 experimental trials yielding 172 trials in total.

During each trial, participants have to adjust the joystick in its central position and press the "fire"-button to initiate the presentation of the picture. Depending on the content of the picture participants have to push or pull the joystick. If the joystick is moved in the correct direction, the picture disappears and the next trial can be initiated by adjusting the joystick in its central position and pressing the "fire"-button. If the joystick is moved sideways or in the wrong direction, the picture remains on the screen until the correct movement is executed.

Single Target Implicit Association Test In order to assess implicit associations between smoking and (a) approach/avoidance and (b) valence (positive/negative), two different ST-IAT [69] are administered. We chose to use a ST-IAT instead of the standard IAT as smoking does not have a "natural" contrast category. For the target category, six smoking-related words are presented. As attribute categories, six approach- and avoidance-related words were selected for the approach-avoid ST-IAT and six positive and negative attributes for the valence ST-IAT (stimuli and experimental design are provided in the Supplementary Material).

Each ST-IAT comprised five blocks: After instructions, the ST-IAT starts with the first block (attribute practice, 12 trials) in which six approach-related/positive and six avoidance-related/negative words are presented randomly and need to be classified by participants using one of two response keys ("E", "I"). In the second (practice combined block, 24 trials) and the third block (test combined block, 48 trials) target as well as attribute words are presented and need to be classified using the two response keys. Participants are instructed to press one key for smoking-related and approach/positive words and the other key for avoidance/negative words (compatible condition). In block 4 (24 trials) and 5 (48 trials), the key of the target category (i.e., smoking) is switched such that smoking and avoidance/negative share one response key (incompatible condition). During each trial, stimuli are presented centrally on a black screen; labels of the attribute categories (German words for "approach" or "positive" versus "avoid" or "negative," written in green ink) and the target category (German word for "smoking", written in white ink) are presented in either the left or right upright corner of the screen. If participants press the wrong key, a red X is presented for 200 ms and the correct response has to be executed before the next trial is initiated. There are two different versions of the ST-IAT: half of the participants receive the compatible-incompatible block order while the other half receives the incompatible-compatible block order. Participants are given the same block order for the approach-avoid and the valence ST-IAT pre- and post-intervention.

Stroop Task The ability to suppress pre-potent responses is assessed by means of a color Stroop task [70] with key press input. Participants are presented four color words as well as colored rectangles and instructed to indicate the print color of the word and the color of the rectangle using one of four computer keys (d = red; f = green; j = blue; k = black). Participants are asked to respond as fast and as accurate as possible. The task comprises three different kinds of trials: (1) congruent (color word and ink color are identical [i.e., red in red ink]); (2) incongruent (color word and ink color are not identical [i.e., red written in green ink]); and (3) control trials (colored rectangles). The Stroop task has a 4 colors (red, green, blue, black) × 3 congruency (congruent, incongruent, control) × 6 repetitions design yielding 72 trials in total. For the incongruent trials, each color word is printed in each incongruent color twice. Trials are presented in fully randomized order. During each trial, stimuli are presented centrally on the screen with key assignments shown in the upper part of the screen (intertrial interval 200 ms). If participants press a wrong key, a red cross is presented (400 ms).

Passive Picture Viewing Task The design and procedure of the task basically follows the procedure used in an earlier study [23]. During the task, psychophysiological measures are assessed in order to measure the motivational significance of smoking-related stimuli: (1) acoustic startle response (EMG; *M. orbicularis oculi*) and (2) facial EMG (*M. corrugator supercilii* and *M. zygomaticus major*).

The task comprises 48 color pictures of four different categories (negative [n = 12], neutral [n = 12], positive [n = 12], smoking-related [n = 12]); smoking-related pictures depict events related to the beginning of smoke intake [71, 72] and are adapted from previous studies ([23] [Exp. 2 A series], [71]) and an unpublished picture set by Mucha and Pauli. Control pictures were taken from the International Affective Picture System (IAPS [73]). Positive and negative pictures are matched according to arousal and absolute valence drawn from IAPS [73]. Numbers of the IAPS pictures are provided in the Supplementary Material (Table S6). Pictures are divided in three blocks with four pictures of each category (i.e., 16 pictures per block).

The general set-up of the experiment is as follows: After the instruction, four practice trials with one picture per category are presented in a fixed order. During three of the practice trials (neutral, smoking-related, positive), an acoustic startle tone is presented 2.5 s, 4.0 s, and 5.5 s after picture onset. Next, three habituation trials without picture presentation follow during which the acoustic startle tone is presented. Subsequently, the experiment starts, consisting of three blocks with 48 pictures per block, each block followed by a short break. Pictures are presented for 7.0–8.0 s (M = 7.5 s), followed by a dark monitor for 16.5 to 25.5 s (M = 21.0 s, intertrial interval). In each block, during three pictures of each category an acoustic startle response is evoked 2.5 s, 4.0 s, and 5.5 s after picture onset. The acoustic startle noise was created in Adobe Audition (Version 1.0) by generating a mono white noise (44,100 Hz, 16 bit) with a duration of 50 ms. It is controlled by the software Presentation (Neurobehavioral Systems), passed through a PreSonus HP4 amplifier, and presented via shielded headphones binaurally (Sennheiser HD 280 Pro) at a constant volume (105 dB). After the final block of picture presentation, participants are asked to rate each picture during a free viewing task in the same order, separated into three blocks. At the beginning, the participants are familiarized with the rating procedure in one trial including a positive picture. The pictures are rated under free viewing conditions regarding valence (1 = "pleasant" to 9 = "unpleasant"), arousal (1 = "relaxed" to 9 = "aroused"), and craving (1 = "not at all" to 9 = "very strong") with rating scales being presented after each picture presentation. The experiment lasts approximately 45 min.

During the passive picture viewing task, the order of picture category (neutral, positive, negative, smoking-related), startle presentation (present, absent), and startle presentation time (2.5 s, 4.0 s, 5.5 s) is pseudorandomized with the following restrictions in each block: no more than two pictures of the same category are presented consecutively, no more than four pictures in a row are presented with a startle noise and no more than two pictures in a row are presented with the same startle probe time. The order of pictures within each picture category was randomized by means of the research randomizer (www.randomizer.org). In total, 14 different orders were created and participants receive the same order pre- and post-intervention.

Psychophysiological assessment Psychophysiological activity is recorded continuously using a 16-channel amplifier (Twente Medical Systems International [TMSi], EJ Oldenzaal, The Netherlands) and the recording software package Polybench 1.30 (TMSi) using a sampling rate of 1024 Hz. The amplifier includes customized channels for measuring various physiological parameters. Facial muscle activity is assessed by three pairs of 2-mm inner-diameter Ag/AgCl electrodes (filled with EMG gel) that are attached to skin sites with centers approximately 1 cm apart (skin was cleaned with Nuprep and alcohol pads). Electrodes for the measurement of *M. corrugator supercilii* activity are attached with adhesive rings and placed above the participant's left eyebrow, for *M. zygomaticus major* activity on the left cheek halfway between mouth and ear tip, and for *M. orbicularis oculi* activity (startle response) beneath the left eye [74, 75]. A wet band on the left wrist served as grounding for all channels.

Intervention

Treatment-as-usual: smoke-free program

As TAU, participants receive the 1-day version of the smoking cessation intervention "smoke-free program" [76] consisting of eight units of 45 min each (units 1–4: preparation of quit attempt; units 5–8: stabilization). Participants receive an individual telephone consultation (15 min) 1 week after the course. The program combines motivational and cognitive behavioral interventions (e.g., motivational interviewing, psychoeducation, cognitive strategies), includes a quit attempt, and follows clinical guidelines. The intervention is manualized and conducted by certified trainers. It is evaluated annually with a mandatory participation of all trainers. The program achieves 6-month abstinence rates between 34 and 38% [77].

Participants are informed that they might potentially experience craving and suffer from depressed mood in the short term after the quit attempt. Participants have the opportunity to discuss these side effects during their individual telephone consultation with their trainer. If participants wish to proceed with their smoking cessation treatment, they are referred to the outpatient treatment center for tobacco dependence of the university hospital Munich after the follow-up assessment.

Add-on: AppBM, Sham training, and TAU only

Contrary to the assessment task, an indirect instruction is used for the training[2]. Response direction depends on the tilt of the pictures (5° to the left/right). During AppBM, all smoking-related pictures are tilted in the direction that is associated with pushing (i.e., avoidance) and all positive pictures in the direction that is associated with pulling (i.e., approach). During Sham training, 50% of the pictures of each category have to be pushed and pulled. In total, 40 pictures are presented (20 smoking-related, 20 positive). Consequently, it is possible to test whether training effects generalize to untrained stimuli as 20 pictures of each category are only presented during the assessment AAT. The procedure is generally the same as in the assessment AAT with the exception that participants are instructed to push and pull the joystick depending on the tilt of the pictures (instruction [push left tilt – pull right tilt versus push right tilt – pull left tilt] is counterbalanced across participants). After 10 practice trials, 240 training trials need to be performed. Participants receive the first training session following the smoke-free program and are asked to perform the training daily for the next 7 days. If participants do not perform the training, email reminders are sent. Participants of the TAU only arm do not receive an additional training.

Assignment of interventions: sequence generation, randomization, allocation concealment, and blinding

Participants who fulfill inclusion criteria, provide informed consent, and complete the baseline assessment for a specific smoke-free course are randomized. Randomization is performed block-wise. The block-wise randomization list with changing block sizes was created at the Munich Centre of Clinical Trials (Münchner Studien Zentrum, MSZ) and is not accessible to study members. Consequently, forthcoming assignments and group allocation are unbeknown to staff responsible for recruitment and enrolment and to trial participants (i.e., allocation concealment). Randomization is performed 1:1:1 centrally. An online tool at the MSZ is used to randomize participants to the study arm (TAU +

[2] An indirect instruction is used for the trainings for two reasons: First, if picture content is task-irrelevant, blinding is more likely to be maintained. Second, it is easier to realize the 50/50 contingency of the Sham training.

AppBM, TAU + Sham, TAU only) according to the predefined randomization list. Randomization is conducted by the PI. Participants of one intervention are randomized after recruitment for a specific course is completed. Participants are informed at the end of the smoke-free intervention whether they receive a training or not. If they receive a training, participants are blind to the training condition as participants of both types of training are provided with the same rationale; however, it is impossible to blind participants randomized to the TAU only condition. Care providers and outcome assessors are also blind to treatment allocation.

Data collection, storage, access, and monitoring

Except the sociodemographic interview and the verification of inclusion criteria, questionnaires as well as the experimental tasks (AAT, ST-IATs, Stroop) are performed on a computer using the experimental software Inquisit® (www.millisecond.com); the passive picture viewing task is implemented through Presentation (Neurobehavioral Systems), and psychophysiological data is recorded with a 16-channel amplifier and the recording software package Polybench 1.30 (TMSi). Participants are assigned a unique code and all assessment data are stored using the code to ensure participant confidentiality. The coding list, all personal information, and the informed consent documents are stored separately from the assessment data in locked cabinets and are only accessible for the principal investigator (PI) and research assistants. The coding list will be destroyed upon completion of data collection so that the data is anonymized; the fully anonymized data will not be destroyed. After publication of study results, it is planned to transfer the fully anonymized data to open data repositories (e.g., OSF). Questionnaire and experimental data are stored on secure servers of the LMU Munich and can only be accessed by authorized personal. All assessors are extensively trained regarding study requirements and standardized assessment; procedures of each assessment and instructions how to store the data are described in detail in a study manual. Data collected on paper will manually be entered in SPSS, self-reported, and experimental and psychophysiological data will automatically be stored electronically. Data integrity of all data will be checked through several means (e.g., correct format [numeric, integer], range check, consistency checks, confirmation of valid values). The PI and authorized study personal have access to the data set(s). There is no data monitoring committee for the present trial; it is also not audited.

Dissemination

Results of the trial are intended for publication in peer-reviewed journals, independent of study outcome. Upon completion of data collection, findings will also be presented at scientific conferences. It is planned to publish at least one article on treatment efficacy and one on working mechanisms. Potentially, data assessed pre-intervention will also be published (e.g., data of experimental tasks).

Retention

In order to prevent drop-out and therefore missing data, several means are implemented. Participants are informed about the necessity of complete data, even in case of non-adherence. At follow-up, only the most important information (abstinence, including biochemical verification, tobacco dependence, approach biases) is assessed to keep the burden low. In addition, participants receive financial reimbursement or course credit for attending the assessments. Prior to each assessment, participants are reminded via email and called if they do not attend the assessment. Additionally, reminders for contacting participants and scheduling appointments are implemented; retention is monitored and methods adapted if retention is unsatisfactory.

Statistical methods

Data reduction

Assessment AAT RTs of incorrect trials will be excluded (joystick moved in wrong direction, change of direction during the trial). Then, RTs < 200 ms and > 2.5SD above the group mean will be excluded [34]. As in previous studies, if participants have > 35% of missing trials, they will be excluded from AAT analyses [27]. Median RTs (in ms) will be used. Then, a bias score for smoking-related pictures will be operationalized as the difference in median response times between push and pull movements for untrained smoking-related pictures (i.e., $push_{smoking} - pull_{smoking}$). As a control, we also create a bias score for positive pictures, which is the difference between the push versus pull responses for untrained positive pictures (i.e., $push_{positive} - pull_{positive}$).

ST-IAT The analytical approach follows the suggestions by Karpinski and Steinman [78]. RTs < 350 ms will be discarded, error responses replaced with the block mean plus a penalty of 400 ms, and participants with > 20% errors excluded from analysis. Only test trials (i.e., blocks 3 and 5) will be used, and the difference between blocks (block 5 [smoking + bad/avoidance] − block 3 [smoking + positive/approach]) will be calculated and divided by the standard deviation of all correct responses of blocks 3 and 5.

Stroop RTs of incorrect trials as well as RTs < 200 ms and > 2.5SD above the group mean will be excluded. Only mean latencies of incongruent trials (in ms) will be

used and corrected for individual response speed by subtracting mean latencies for color naming (i.e., RT incongruent trials – RT control trials).

Psychophysiology

The signals will be analyzed using the software Autonomic Nervous System Laboratory (ANSLAB) version 2.6 [79]. Facial EMG is measured. All EMG data will be preprocessed using a 28-Hz high-pass filter, 50-Hz notch filter, rectification, low pass filtering (500 Hz), and 50 ms (acoustic startle), and 150 ms (*M. zygomaticus* and corrugator) moving average filter.

Acoustic startle (*M. orbicularis*) Scoring of the startle responses will be computer-assisted and done by two independent raters blind to experimental condition. Responses will be scored as valid, invalid (e.g., spontaneous eye blink during baseline, during probe and response onset, excessive noise, movement artifacts), and zero (e.g., startle peak does not clearly exceed the overall variability of the EMG signal) following recommendations by Blumenthal et al.. For valid trials, the variable of interest is response amplitude, which is defined as the difference between the maximal EMG value 20–200 ms after probe onset relative to the average EMG value during a 50-ms pre-probe baseline period [75]. For analyses, magnitude will be determined per condition (i.e., non-response trials are included). If necessary, inter-individual differences in blink magnitudes and EMG data will be standardized for each subject prior to analyses.

***M. zygomaticus* and corrugator** The muscle activity will be calculated as the difference between mean EMG response during picture presentation (shortest presentation time 7 s) minus a 1-s baseline period. EMG is computed as the average for each experimental period of interest (in μV). All trials will be considered for EMG analyses. The mean activity will be calculated for each the *M. zygomaticus* and the *M. corrugator* per category and participant.

Data analyses

Baseline characteristics We will examine group differences in baseline characteristics such as demographics, smoking-related and psychological variables, and performance in the Stroop task. Distributional properties of the measures will be examined and, if appropriate, considered during analyses.

Dropout Dropout rates will be compared between groups; it will also be examined whether completers and non-completers differ as to baseline characteristics.

Smoking- and health-related information Following SPIRIT guidelines [48], an ITT approach will be applied, that is, all randomized participants will be included in analyses and retained in the group in which they were randomized, irrespective of adherence to the protocol (exception: participant died during study participation; moved to unknown address, see [62]). We expect that TAU + AppBM is more effective in increasing long-term abstinence than both control groups. The primary outcome (abstinence at the 6-month follow-up) will be analyzed using a logistic regression (including appropriate covariates). Secondary outcomes, assessed pre-intervention, post-intervention, and at the 6-month follow-up (tobacco dependence [CDS-12]; daily cigarette consumption, CO value), will be analyzed using multilevel modeling with group allocation, time, and their interaction terms as predictors. Time is dummy-coded for the comparisons pre-intervention versus post-intervention and pre-intervention versus follow-up. Multilevel models will also be performed with secondary outcomes assessed at pre-intervention and post-intervention (craving [QSU-brief], BSI).

Experimental tasks Data of all experimental tasks serve as secondary outcomes. These indices are also analyzed as mediators to explain the effect of the training on clinical outcome. Regarding the AAT, it is expected that participants who receive TAU + AppBM will show an increase in the avoidance bias of smoking-related pictures as measured with the AAT compared to both control groups. Therefore, the bias score for untrained smoking-related pictures serves as dependent variable. The planned analyses for the outcomes of experimental tasks are in parallel to those of the other secondary outcomes; the multilevel modeling with the bias scores (AAT), the D score (ST-IATs), and EMGs as outcomes and with time, group, and their interaction as predictors.

Mediation

Given the rationale of CBM that effects of the training on outcome are mediated by a change in approach biases, it is hypothesized that the combination of TAU + AppBM should increase avoidance biases for untrained smoking-related stimuli, which in turn should be associated with higher abstinence rates at follow-up. This hypothesis is formulated as a typical mediation model with X: Group, M: Change in the bias score for untrained smoking-related pictures between the pre- and post-intervention assessments, and Y: Abstinence. The model will be specified in the framework of structural equation modeling with weighted least square mean and variance adjusted estimator (WLSMV) for the dichotomous outcome; mediation analysis will be restricted to the per-protocol sample (see Additional Analysis) as a robust

estimator [80]. We are specifically interested in the direct effect (group on abstinence) as well as the indirect effect(s) via the change in the bias score(s). The indirect effects will be tested with bootstrapping with a sufficient number of iterations.

However, it has not systematically been investigated whether effects of the training on outcome are (partially) mediated by changing other components of information processing. Therefore, potential alternative processes will also be explored: (1) devaluation of smoking-related stimuli as assessed with the valence ST-IAT [47]. If effects of TAU + AppBM on abstinence are mediated by a devaluation of smoking-related stimuli, attitudes in the valence ST-IAT should become more negative from pre- to post-intervention. (2) Increase of tobacco-avoidance associations [27, 43]. If effects of TAU + AppBM on abstinence are mediated by an increase of tobacco-avoidance associations as assessed with the approach-avoid ST-IAT, tobacco-avoidance associations should increase from pre- to post-intervention. (3) Decrease in motivational orientation (acoustic startle) and/or valence (facial EMG). If effects of TAU + AppBM on abstinence are mediated by a decrease of the motivational significance of smoking-related stimuli, TAU + AppBM should result in a stronger ASR, less activity of the zygomatic muscle, and increased activity of the corrugator muscle when viewing smoking-related stimuli from pre- to post-intervention. Extra mediators will be added to the above-described mediation model. The three alternative mediators, namely (1) devaluation of smoking-related stimuli, (2) increase of tobacco-avoidance associations, and (3) decrease in motivational significance will be added simultaneously or sequentially. The same estimation approach will be used to the mediation analysis described above.

Additional analyses

As an exploratory analysis, multilevel models for secondary outcomes (tobacco dependence, CPD) will be estimated with relapse (yes/no) as a moderator on the treatment effect. Specifically, we will test the time × group × relapse interaction in order to clarify whether participants of the TAU + AppBM group who relapsed after treatment will smoke less compared to participants of the TAU + Sham and TAU only groups.

Additionally, per-protocol analyses will be performed on completers. We will use (at least) two criteria for the training groups (TAU + AppBM, TAU + Sham): (a) strict criterion, with which completers have to have completed at least five training sessions [81] and have participated in all three assessments (i.e., pre-intervention, post-intervention, 6-month follow-up); (b) “generous” criterion, which regards participants as completers who have completed at least one training session [34] and have participated in all three assessments. Sensitivity analyses are planned to clarify if our results are robust to the different criteria of completers. For the TAU only group, participants have to complete all assessments to be counted as completer. No interim analyses will be conducted.

Moderation analyses

It has been proposed in dual-process models that individual differences regarding controlled processes and impulsivity moderate the interplay between impulsive processes and substance use [11], an assumption that has been corroborated by empirical evidence [82–84]. Therefore, it will be investigated whether effects of the training on outcome are moderated by individual differences in inhibitory capacities (Stroop) and impulsivity (BIS-15). It is assumed that the training is more effective in individuals with lower inhibitory capacities and higher impulsivity.

Discussion

This paper describes the protocol of a randomized-controlled, double-blind clinical trial which primarily aims to investigate the efficacy of AppBM as an add-on to a CBT-based smoking cessation intervention. Additionally, potential working mechanisms of AppBM are investigated, namely, change in smoking approach bias (AAT), devaluation of smoking-related stimuli (valence ST-IAT), smoking-avoidance associations (approach-avoid ST-IAT), and psychophysiological measures of the motivational significance of smoking-related stimuli.

Although evidence-based interventions for smoking cessation are available, long-term abstinence still is the exception rather than the norm. Consequently, there is a need to develop interventions that improve outcome. Targeting processes that are not considered in current treatments represent a possible starting point. Based on the prominent role that implicit information processing biases are ascribed in substance dependence [10, 11], past research tested the efficacy of CBM trainings aiming at directly manipulating these biases [37, 84]. Specifically, AppBM has shown robust and clinically relevant effects in improving long-term abstinence in samples of abstinent inpatients with alcohol use disorder [26–28]. However, evidence regarding the efficacy of AppBM in smoking has been inconsistent [32–34, 85]. Moreover, most studies in smoking have been focused on nicotine consumption [32, 85], but only one study included long-term abstinence as a clinical outcome [34]. Therefore, it remains unresolved whether targeting approach biases in smoking can improve long-term clinical outcome. As large-scale clinical studies in representative samples of adult smokers in regular care are lacking, the aim of the present study is to fill this gap. If effective, AppBM holds

the advantage that it is easy to deliver, cost-effective, and has no adverse effects. Increasing long-term abstinence from smoking would not only yield substantial health benefits [3, 4], but would also decrease the direct and indirect costs associated smoking. If not effective, the question why divergent findings emerge in smoking versus other substance use disorders needs to be addressed in future research. The trial represents an important step in advancing our knowledge on implicit biases and their modification in smoking. If approach biases can successfully be modified, but no effects on clinical outcome emerge, one might question whether approach biases are a relevant characteristic of smoking behavior.

The second goal of the trial is to improve our understanding *how* AppBM works in order to test the theoretical rationale of AppBM. As outlined in the introduction, the central tenet of CBM is that if the bias of interest is changed successfully, effects on symptoms should emerge [42]. Consequently, it is crucial to investigate whether the intended bias change (i.e., smoking approach bias) has been achieved [41]. However, positive effects on outcome have been observed although effects of the training on outcome were not mediated by a bias change [27, 32]. This raises the possibility that AppBM affects other biases, for example, substance-avoidance associations [27, 44], or works by reducing the positive valence of substance-related stimuli [45, 86]; however, the systematic investigation of effects of AppBM on different components of information processing is still pending. The present trial aims to identify information processing biases susceptible to change and to test whether changes predict clinical outcome. As a TAU only arm is included, the trial permits to investigate to what extent the smoking cessation intervention affects implicit information processing. Consequently, the present trial informs theoretical accounts on implicit biases in smoking and provides novel insights into potential mechanisms of change. If effects of AppBM on outcome should be mediated by another process, it seems worthwhile to directly address this process in future trainings and to investigate whether targeting the alternative mechanisms is more effective than AppBM.

Strengths of the present trial include a sound design (double-blind, randomized placebo-controlled) and the inclusion of a TAU only condition. Furthermore, a representative sample of adult smokers is recruited which increases generalizability of findings. As several implicit biases are assessed, this is the first study that allows the investigation of different potential mediators thereby advancing our knowledge on mechanisms of change. Potential limitations of the current trial include risk of substantial drop-out, non-adherence to the protocol (e.g., non-usage of training), and protocol violation (e.g., NRT during trial participation). Due to the Corona pandemic, the study had to be stopped temporarily; therefore, there is a risk that the intended sample size of 336 participants cannot be reached, which would reduce statistical power.

Trial status

The first participants were enrolled in November 2019. Originally, we aimed to recruit the final sample size within around 2 years; however, due to the Corona pandemic and interim closing of university buildings, recruitment will be delayed at least until spring 2023.

Abbreviations

AAT: Approach-Avoidance Task; AppBM: Approach Bias Modification; BIS-15: Barratt Impulsiveness Scale-15; BSI: Brief Symptom Inventory; CBM: Cognitive Bias Modification; CBT: Cognitive Behavioral Therapy; CDS-12: Cigarette Dependence Scale-12; CO: Carbon monoxide; CPD: Cigarettes per day; DSM-5: Diagnostic and Statistical Manual for Mental Disorders; EMG: Electromyography; FTND: Fagerstrøm Test for Nicotine Dependence; FU: Follow-up; GMA: Growth-Modeling Analysis; IAPS: International Affective Picture SystemIATImplicit Association Test; MINI: Mini International Neuropsychiatric Interview; MSZ: Münchner Studien Zentrum; NRT: Nicotine Replacement Therapy; PPM: Parts per million; QSU-brief: Brief Questionnaire of Smoking Urges; RS: Russell Standard; (ST)-IAT: (Single Target) Implicit Association Task; TAU: Treatment-as-usual; WSQ: Web Screening Questionnaire for Common Mental Disorders

Supplementary Information

The online version contains supplementary material available at https://doi.org/10.1186/s13063-022-06155-6.

Additional file 1. SPIRIT 2013 Checklist: Recommended items to address in a clinical trial protocol and related documents.

Additional file 2: Table S1. Counterbalancing of Instructions (AAT, IAT) and Order of IATs. **Table S2.** Ratings of Smoking-Related and Positive Pictures for Each Picture Set Used in the Approach-Avoidance Task (AAT). **Table S3.** Ratings of Smoking-Related and Positive Pictures Used in the Approach-Avoidance Task (AAT). **Table S4.** Design of the Single Target Implicit Association Test (ST-IAT) for the Order Compatible-Incompatible. **Table S5.** Design of the Single Target Implicit Association Test (ST-IAT) for the Order Incompatible-Compatible. **Table S6.** Picture Numbers (IAPS) of all Control Stimuli.

Authors' contributions

CEW acquired funding, designed the study, and wrote the manuscript. KT performed the analytic calculations as well as the numerical simulations and will support data analyses. PS provided feedback on statistical methods and will support statistical analyses. MHW designed and implemented the Passive Picture Viewing task. GGW supported the inclusion of physiological measures (including later analyses). TE provided feedback to the theoretical rationale, study design, and measures used. TR provided feedback on the study design and supervision. All authors read and approved the final manuscript.

Funding

Open Access funding enabled and organized by Projekt DEAL. This study was supported by a grant of the German Research Foundation (Deutsche Forschungsgemeinschaft [DFG]) granted to Charlotte E. Wittekind (WI4856/3-1). The funding agency has no influence on the design of the study, collection, analysis, and interpretation of data, on writing the report(s), and the decision to submit results for publication.

Availability of data and materials

The datasets of the current trial will be made publicly available after an embargo period or after publication of the major findings. Before publication, data will be available from the first author on request.

Declarations

Ethics approval and consent to participate
The trial is registered at the German Clinical Trials Register (DRKS00019221). The trial and the informed consent have been approved by the ethics committee of the Department of Psychology and Education at LMU (23_Wittekind_c_2019). All participants provide written informed consent prior to participation. Important modifications to the trial (e.g., inclusion criteria, sample size) will be communicated to the Clinical Trials Register and the ethics committee.

Consent for publication
Not applicable.

Competing interests
CEW, KT, PS, MHW, GGW, and TE: The authors declare that they have no competing interests. TR: Pfizer Pharma, Johnson & Johnson: talks and advisory boards. Boehringer Ingelheim, Janssen-Cilag: talks.

Author details
[1]Department of Psychology, LMU Munich, Leopoldstraße 13, 80802 Munich, Germany. [2]Department of Psychology I, Biological Psychology, Clinical Psychology, and Psychotherapy, University of Würzburg, Marcusstr. 9-11, 97070 Würzburg, Germany. [3]Department of Psychiatry and Psychotherapy, LMU Munich, University Hospital, Nußbaumstr. 7, 80336 Munich, Germany.

Received: 20 August 2021 Accepted: 7 March 2022
Published online: 21 March 2022

References
1. US Department of Health and Human Services. The health consequences of smoking: 50 years of progress. A report of the Surgeon General. Atlanta: U.S. Department of Health and Human Services, Centers for Disease Control and Prevention, National Center for Chronic Disease Prevention and Health Promotion, Office on Smoking and Health; 2014.
2. Reitsma MB, Fullman N, Ng M, Salama JS, Abajobir A, Abate KH, et al. Smoking prevalence and attributable disease burden in 195 countries and territories, 1990-2015: a systematic analysis from the global burden of disease study 2015. Lancet. 2017;389(10082):1885–906. https://doi.org/10.1016/S0140-6736(17)30819-X.
3. Doll R, Peto R, Boreham J, Sutherland I. Mortality in relation to smoking: 50 years' observations on male British doctors. BMJ. 2004;328(7455):1519. https://doi.org/10.1136/bmj.38142.554479.AE.
4. Jha P, Ramasundarahettige C, Landsman V, Rostron B, Thun M, Anderson RN, et al. 21st-century hazards of smoking and benefits of cessation in the United States. N Engl J Med. 2013;368(4):341–50. https://doi.org/10.1056/NEJMsa1211128.
5. Arbeitsgemeinschaft der Wissenschaftlichen Medizinischen Fachgesellschaften (AWMF). S3-Leitlinie "Rauchen und Tabakabhängigkeit: Screening, Diagnostik und Behandlung". 2021. Available from https://www.awmf.org/uploads/tx_szleitlinien/076-006l_S3_Rauchen-_Tabakabhaengigkeit-Screening-Diagnostik-Behandlung_2021-03.pdf.
6. National Institute for Health and Care Excellence [NICE]. Stop smoking interventions and services. 2018. Available from: https://www.guidelines.co.uk/smoking-cessation/nice-smoking-cessation-guideline/454141.article.
7. Fiore M, Jaén C, Baker T, et al. Treating tobacco use and dependence: 2008 update. Clinical Practice Guideline. Rockville, MD: U.S. Department of Health and Human Services. Public Health Service. May 2008. Available from: https://www.ncbi.nlm.nih.gov/books/NBK63952/.
8. Mottillo S, Filion KB, Bélisle P, Joseph L, Gervais A, O'Loughlin J, et al. Behavioural interventions for smoking cessation: a meta-analysis of randomized controlled trials. Eur Heart J. 2009;30(6):718–30. https://doi.org/10.1093/eurheartj/ehn552.
9. Dobbie F, Hiscock R, Leonardi-Bee J, Murray S, Shahab L, Aveyard P, et al. Evaluating long-term outcomes of NHS stop smoking services (ELONS): a prospective cohort study. Health Technol Assess. 2015;19(95):9–155. https://doi.org/10.3310/hta19950.
10. Deutsch R, Strack F. Reflective and impulsive determinants of addictive behavior. In: Wiers RW, Stacy AW, editors. Handbook of implicit cognition and addiction. Thousand Oaks: Sage; 2006. p. 45–57. https://doi.org/10.4135/9781412976237.n4.
11. Wiers RW, Bartholow BD, van den Wildenberg E, Thush C, Engels RC, Sher KJ, et al. Automatic and controlled processes and the development of addictive behaviors in adolescents: a review and a model. Pharmacol Biochem Behav. 2007;86(2):263–83. https://doi.org/10.1016/j.pbb.2006.09.021.
12. Bechara A. Decision making, impulse control and loss of willpower to resist drugs: a neurocognitive perspective. Nat Neurosci. 2005;8(11):1458–63. https://doi.org/10.1038/nn1584.
13. Wiers RW, Gladwin TE, Hofmann W, Salemink E, Ridderinkhof KR. Cognitive bias modification and cognitive control training in addiction and related psychopathology: mechanisms, clinical perspectives, and ways forward. Clin Psychol Sci. 2013;1(2):192–212. https://doi.org/10.1177/2167702612466547.
14. Robinson TE, Berridge KC. The neural basis of drug craving: an incentive-sensitization theory of addiction. Brain Res Rev. 1993;18(3):247–91. https://doi.org/10.1016/0165-0173(93)90013-P.
15. Everitt BJ, Belin D, Economidou D, Pelloux Y, Dalley JW, Robbins TW. Neural mechanisms underlying the vulnerability to develop compulsive drug-seeking habits and addiction. Philos Trans R Soc B Biol Sci. 2008;363(1507):3125–35. https://doi.org/10.1098/rstb.2008.0089.
16. Koob GF, Le Moal M. Addiction and the brain antireward system. Annu Rev Psychol. 2008;59(1):29–53. https://doi.org/10.1146/annurev.psych.59.103006.093548.
17. Stacy AW, Wiers RW. Implicit cognition and addiction: a tool for explaining paradoxical behavior. Annu Rev Clin Psychol. 2010;6(1):551–75. https://doi.org/10.1146/annurev.clinpsy.121208.131444.
18. Cox WM, Fadardi JS, Pothos EM. The addiction-stroop test: theoretical considerations and procedural recommendations. Psychol Bull. 2006;132(3):443–76. https://doi.org/10.1037/0033-2909.132.3.443.
19. Field M, Cox WM. Attentional bias in addictive behaviors: a review of its development, causes, and consequences. Drug Alcohol Depend. 2008;97(1-2):1–20. https://doi.org/10.1016/j.drugalcdep.2008.03.030.
20. Machulska A, Zlomuzica A, Adolph D, Rinck M, Margraf J. "A cigarette a day keeps the goodies away": smokers show automatic approach tendencies for smoking—but not for food-related stimuli. PLoS One. 2015;10(2):e0116464. https://doi.org/10.1371/journal.pone.0116464.
21. Wiers CE, Kühn S, Javadi AH, Korucuoglu O, Wiers RW, Walter H, et al. Automatic approach bias towards smoking cues is present in smokers but not in ex-smokers. Psychopharmacology. 2013;229(1):187–97. https://doi.org/10.1007/s00213-013-3098-5.
22. Rinck M, Becker ES. Approach and avoidance in fear of spiders. J Behav Ther Exp Psychiatry. 2007;38(2):105–20. https://doi.org/10.1016/j.jbtep.2006.10.001.
23. Geier A, Mucha RF, Pauli P. Appetitive nature of drug cues confirmed with physiological measures in a model using pictures of smoking. Psychopharmacology. 2000;150(3):283–91. https://doi.org/10.1007/s002130000404.
24. Lechner WV, Grant DMM, Meier E, Mills AC, Judah MR, Dempsey JP. The influence of stress on the affective modulation of the startle response to nicotine cues. Appl Psychophysiol Biofeedback. 2014;39(3-4):279–85. https://doi.org/10.1007/s10484-014-9266-5.
25. Drobes DJ, Tiffany ST. Induction of smoking urge through imaginal and in vivo procedures: physiological and self-report manifestations. J Abnorm Psychol. 1997;106(1):15–25. https://doi.org/10.1037/0021-843X.106.1.15.
26. Rinck M, Wiers RW, Becker ES, Lindenmeyer J. Relapse prevention in abstinent alcoholics by Cognitive Bias Modification: clinical effects of combining Approach Bias Modification and Attention Bias Modification. J Consult Clin Psycholoy. 2018;86(12):1005–16. https://doi.org/10.1037/ccp0000321.
27. Wiers RW, Eberl C, Rinck M, Becker ES, Lindenmeyer J. Retraining automatic action tendencies changes alcoholic patients' approach bias for alcohol and improves treatment outcome. Psychol Sci. 2011;22(4):490–7. https://doi.org/10.1177/0956797611400615.
28. Eberl C, Wiers RW, Pawelczack S, Rinck M, Becker ES, Lindenmeyer J. Approach bias modification in alcohol dependence: do clinical effects replicate and for whom does it work best? Dev Cogn Neurosci. 2013;4:38–51. https://doi.org/10.1016/j.dcn.2012.11.002.
29. Manning V, Staiger PK, Hall K, Garfield JBB, Flaks G, Leung D, et al. Cognitive bias modification training during inpatient alcohol detoxification reduces early relapse: a randomized controlled trial. Alcohol Clin Exp Res. 2016;40(9):2011–9. https://doi.org/10.1111/acer.13163.
30. Manning V, Garfield JBB, Staiger PK, Lubman DI, Lum JAG, Reynolds J, et al. Effect of Cognitive Bias Modification on early relapse among adults

undergoing inpatient alcohol withdrawal treatment: a randomized clinical trial. JAMA Psychiatry. 2021;78(2):133–40. https://doi.org/10.1001/jamapsychiatry.2020.3446.
31. Wittekind CE, Feist A, Schneider BC, Moritz S, Fritzsche A. The approach-avoidance task as an online intervention in cigarette smoking: a pilot study. J Behav Ther Exp Psychiatry. 2015;46:115–20. https://doi.org/10.1016/j.jbtep.2014.08.006.
32. Machulska A, Zlomuzica A, Rinck M, Assion HJ, Margraf J. Approach bias modification in inpatient psychiatric smokers. J Psychiatr Res. 2016;76:44–51. https://doi.org/10.1016/j.jpsychires.2015.11.015.
33. Baird SO, Rinck M, Rosenfield D, Davis ML, Fisher JR, Becker ES, et al. Reducing approach bias to achieve smoking cessation: a pilot randomized placebo-controlled trial. Cognit Ther Res. 2017;41(4):662–70. https://doi.org/10.1007/s10608-017-9835-z.
34. Wittekind CE, Reibert E, Takano K, Ehring T, Pogarell O, Rüther T. Approach-avoidance modification as an add-on in smoking cessation: a randomized controlled study. Behav Res Ther. 2019;114:35–43. https://doi.org/10.1016/j.brat.2018.12.004.
35. Kong G, Larsen H, Cavallo DA, Becker D, Cousijn J, Salemink E, et al. Re-training automatic action tendencies to approach cigarettes among adolescent smokers: a pilot study. Am J Drug Alcohol Abuse. 2015;41(5):425–32. https://doi.org/10.3109/00952990.2015.1049492.
36. Cristea IA, Kok RN, Cuijpers P. The effectiveness of Cognitive Bias Modification interventions for substance addictions: a meta-analysis. PLoS ONE. 2016;11(9):e0162226. https://doi.org/10.1371/journal.pone.0162226.
37. Boffo M, Zerhouni O, Gronau QF, van Beek RJJ, Nikolaou K, Marsman M, et al. Cognitive Bias Modification for behavior change in alcohol and smoking addiction: Bayesian meta-analysis of individual participant data. Neuropsychol Rev. 2019;29(1):52–78. https://doi.org/10.1007/s11065-018-9386-4.
38. MacLeod C, Rutherford E, Campbell L, Ebsworthy G, Holker L. Selective attention and emotional vulnerability: assessing the causal basis of their association through the experimental manipulation of attentional bias. J Abnorm Psychol. 2002;111(1):107–23. https://doi.org/10.1037/0021-843X.111.1.107.
39. Jones EB, Sharpe L. Cognitive bias modification: a review of meta-analyses. J Affect Disord. 2017;223:175–83. https://doi.org/10.1016/j.jad.2017.07.034.
40. Kakoschke N, Kemps E, Tiggemann M. Approach bias modification training and consumption: a review of the literature. Addict Behav. 2017;64:21–8. https://doi.org/10.1016/j.addbeh.2016.08.007.
41. MacLeod C, Clarke PJF. The attentional bias modification approach to anxiety intervention. Clin Psychol Sci. 2015;3(1):58–78. https://doi.org/10.1177/2167702614560749.
42. Grafton B, MacLeod C, Rudaizky D, Holmes EA, Salemink E, Fox E, et al. Confusing procedures with process when appraising the impact of cognitive bias modification on emotional vulnerability. Br J Psychiatry. 2017;211(5):266–71. https://doi.org/10.1192/bjp.bp.115.176123.
43. Wiers RW, Rinck M, Kordts R, Houben K, Strack F. Retraining automatic action-tendencies to approach alcohol in hazardous drinkers. Addiction. 2010;105(2):279–87. https://doi.org/10.1111/j.1360-0443.2009.02775.x.
44. Gladwin TE, Rinck M, Eberl C, Becker ES, Lindenmeyer J, Wiers RW. Mediation of cognitive bias modification for alcohol addiction via stimulus-specific alcohol avoidance association. Alcohol Clin Exp Res. 2015;39(1):101–7. https://doi.org/10.1111/acer.12602.
45. Veling H, Holland RW, van Knippenberg A. When approach motivation and behavioral inhibition collide: behavior regulation through stimulus devaluation. J Exp Soc Psychol. 2008;44(4):1013–9. https://doi.org/10.1016/j.jesp.2008.03.004.
46. Veling H, Lawrence NS, Chen Z, van Koningsbruggen GM, Holland RW. What is trained during food go/no-go training? A review focusing on mechanisms and a research agenda. Curr Addict Reports. 2017;4(1):35–41. https://doi.org/10.1007/s40429-017-0131-5.
47. Macy JT, Chassin L, Presson CC, Sherman JW. Changing implicit attitudes toward smoking: results from a web-based approach-avoidance practice intervention. J Behav Med. 2015;38(1):143–52. https://doi.org/10.1007/s10865-014-9585-2.
48. Chan AW, Tetzlaff JM, Gøtzsche PC, Altman DG, Mann H, Berlin JA, et al. SPIRIT 2013 explanation and elaboration: guidance for protocols of clinical trials. BMJ. 2013;346(jan08 15):e7586. https://doi.org/10.1136/bmj.e7586.
49. Heatherton TF, Kozlowski LT, Frecker RC, Fagerstrom KO. The Fagerstrom Test for Nicotine Dependence: a revision of the Fagerstrom Tolerance Questionnaire. Br J Addict. 1991;86(9):1119–27. https://doi.org/10.1111/j.1360-0443.1991.tb01879.x.
50. American Psychiatric Association (APA). Diagnostic and Statistical Manual of Mental Disorders: DSM-5. Arlington: American Psychiatric Association; 2013.
51. Green P, Macleod CJ. SIMR: an R package for power analysis of generalized linear mixed models by simulation. Methods Ecol Evol. 2016;7(4):493–8. https://doi.org/10.1111/2041-210X.12504.
52. Feingold A. Effect sizes for growth-modeling analysis for controlled clinical trials in the same metric as for classical analysis. Psychol Methods. 2009;14(1):43–53. https://doi.org/10.1037/a0014699.
53. Sheehan DV. Mini International Neuropsychiatric Interview 7.0.2. Jacksonville: Medical Outcomes Systems; 2016.
54. Donker T, van Straten A, Marks I, Cuijpers P. A brief web-based screening questionnaire for common mental disorders: development and validation. J Med Internet Res. 2009;11(3):e19. https://doi.org/10.2196/jmir.1134.
55. Derogatis LR. Brief Symptom Inventory (BSI), administration, scoring and procedures manual. 3rd ed. Minneapolis: National Computer Services; 1993.
56. Franke GH. Brief Symptom Inventory von L. R. Derogatis (Kurzform der SCL-90 - R) - Deutsche Version. Göttingen: Beltz Test GmbH; 2000.
57. De Meneses-Gaya IC, Zuardi AW, Loureiro SR, Alexandre J, Crippa DS. Psychometric properties of the Fagerström Test for Nicotine Dependence. J Bras Pneumol. 2009;35(1):73–82. https://doi.org/10.1590/S1806-37132009000100011.
58. Leung D, Staiger PK, Hayden M, Lum JAG, Hall K, Manning V, et al. Meta-analysis of the relationship between impulsivity and substance-related cognitive biases. Drug Alcohol Depend. 2017;172:21–33. https://doi.org/10.1016/j.drugalcdep.2016.11.034.
59. Meule A, Vögele C, Kübler A. Psychometrische Evaluation der deutschen Barratt Impulsiveness Scale - Kurzversion (BIS-15) [Psychometric evaluation of the German Barratt Impulsiveness Scale - Short Version (BIS-15)]. Diagnostica. 2011;57(3):126–33. https://doi.org/10.1026/0012-1924/a000042.
60. Spinella M. Normative data and a short form of the Barratt Impulsiveness Scale. Int J Neurosci. 2007;117(3):359–68. https://doi.org/10.1080/00207450600588881.
61. Miyake A, Friedman NP, Emerson MJ, Witzki AH, Howerter A, Wager TD. The unity and diversity of executive functions and their contributions to complex "frontal lobe" tasks: a latent variable analysis. Cogn Psychol. 2000;41(1):49–100. https://doi.org/10.1006/cogp.1999.0734.
62. West R, Hajek P, Stead L, Stapleton J. Outcome criteria in smoking cessation trials: proposal for a common standard. Addiction. 2005;100(3):299–303. https://doi.org/10.1111/j.1360-0443.2004.00995.x.
63. Etter J-F, Le Houezec J, Perneger TV. A self-administered questionnaire to measure dependence on cigarettes: the cigarette dependence scale. Neuropsychopharmacology. 2003;28(2):359–70. https://doi.org/10.1038/sj.npp.1300030.
64. Cox LS, Tiffany ST, Christen AG. Evaluation of the brief questionnaire of smoking urges (QSU-brief) in laboratory and clinical settings. Nicotine Tob Res. 2001;3(1):7–16. https://doi.org/10.1080/14622200020032051.
65. Oliver JA, Drobes DJ. Visual search and attentional bias for smoking cues: the role of familiarity. Exp Clin Psychopharmacol. 2012;20(6):489–96. https://doi.org/10.1037/a0029519.
66. Khazaal Y, Zullino D, Billieux J. The Geneva smoking pictures: development and preliminary validation. Eur Addict Res. 2012;18(3):103–9. https://doi.org/10.1159/000335083.
67. Kopetz C, MacPherson L, Mitchell AD, Houston-Ludlam AN, Wiers RW. A novel training approach to activate alternative behaviors for smoking in depressed smokers. Exp Clin Psychopharmacol. 2017;25(1):50–60. https://doi.org/10.1037/pha0000108.
68. Ferentzi H, Scheibner H, Wiers R, Becker ES, Lindenmeyer J, Beisel S, et al. Retraining of automatic action tendencies in individuals with obesity: a randomized controlled trial. Appetite. 2018;126:66–72. https://doi.org/10.1016/j.appet.2018.03.016.
69. Wigboldus DHJ, Holland RW, van Knippenberg A. Single target implicit associations. 2004. Unpublished manuscript.
70. Stroop JR. Studies of interference in serial and verbal reactions. J Exp Psychol. 1935;18(6):643–62. https://doi.org/10.1037/h0054651.
71. Mucha RF, Pauli P, Weber M, Winkler M. Smoking stimuli from the terminal phase of cigarette consumption may not be cues for smoking in healthy smokers. Psychopharmacology. 2008;201(1):81–95. https://doi.org/10.1007/s00213-008-1249-x.
72. Stippekohl B, Winkler M, Mucha RF, Pauli P, Walter B, Vaitl D, et al. Neural responses to BEGIN-and END-stimuli of the smoking ritual in nonsmokers,

nondeprived smokers, and deprived smokers. Neuropsychopharmacology. 2010;35(5):1209–25. https://doi.org/10.1038/npp.2009.227.
73. Lang PJ, Bradley MM, Cuthbert BN. International affective picture system (IAPS): affective ratings of pictures and instruction manual. Technical Report A-8. Gainesville: The Center for Research in Psychophysiology, University of Florida; 2008.
74. Fridlund AJ, Cacioppo JT. Guidelines for human electromyographic research. Psychophysiology. 1986;23(5):567–89. https://doi.org/10.1111/j.1469-8986.1986.tb00676.x.
75. Blumenthal TD, Cuthbert BN, Filion DL, Hackley S, Lipp OV, Van Boxtel A. Committee report: guidelines for human startle eyeblink electromyographic studies. Psychophysiology. 2005;42(1):1–15. https://doi.org/10.1111/j.1469-8986.2005.00271.x.
76. IFT-Gesundheitsförderung. Das Rauchfrei Programm Trainermanual. 2013.
77. Kröger C, Gröll J. Das Rauchfrei Programm: Jahresbericht 2018. 2018. Available from: https://rauchfrei-programm.de/images/Rauchfrei_Jahresbericht_2018.pdf.
78. Karpinski A, Steinman RB. The single category implicit association test as a measure of implicit social cognition. J Pers Soc Psychol. 2006;91(1):16–32. https://doi.org/10.1037/0022-3514.91.1.16.
79. Blechert J, Peyk P, Liedlgruber M, Wilhelm FH. ANSLAB: integrated multichannel peripheral biosignal processing in psychophysiological science. Behav Res Methods. 2016;48(4):1528–45. https://doi.org/10.3758/s13428-015-0665-1.
80. Rosseel Y. Lavaan: An R package for structural equation modeling. J Stat Softw. 2012;48(2):1–36. https://doi.org/10.18637/jss.v048.i02.
81. Eberl C, Wiers RW, Pawelczack S, Rinck M, Becker ES, Lindenmeyer J. Implementation of approach bias re-training in alcoholism-how many sessions are needed? Alcohol Clin Exp Res. 2014;38(2):587–94. https://doi.org/10.1111/acer.12281.
82. Houben K, Wiers RW. Response inhibition moderates the relationship between implicit associations and drinking behavior. Alcohol Clin Exp Res. 2009;33(4):626–33. https://doi.org/10.1111/j.1530-0277.2008.00877.x.
83. Peeters M, Wiers RW, Monshouwer K, van de Schoot R, Janssen T, Vollebergh WAM. Automatic processes in at-risk adolescents: the role of alcohol-approach tendencies and response inhibition in drinking behavior. Addiction. 2012;107(11):1939–46. https://doi.org/10.1111/j.1360-0443.2012.03948.x.
84. Mühlig S, Paulick J, Lindenmeyer J, Rinck M, Cina R, Wiers RW. Applying the "cognitive bias modification" concept to smoking cessation - A systematic review. Sucht. 2016;62(6):333–54. https://doi.org/10.1024/0939-5911/a000454.
85. Wittekind CE, Lüdecke D, Cludius B. Web-based approach bias modification in smokers: a randomized-controlled study. Behav Res Ther. 2019;116:52–60. https://doi.org/10.1016/j.brat.2018.12.003.
86. Wiers CE, Stelzel C, Gladwin TE, Park SQ, Pawelczack S, Gawron CK, et al. Effects of cognitive bias modification training on neural alcohol cue reactivity in alcohol dependence. Am J Psychiatry. 2015;172(4):335–43. https://doi.org/10.1176/appi.ajp.2014.13111495.
87. Stewart J, de Wit H, Eikelboom R. Role of unconditioned and conditioned drug effects in the self-administration of opiates and stimulants. Psychol Rev. 1984;91(2):251–68. https://doi.org/10.1037/0033-295X.91.2.251.
88. Mucha RF, Geier A, Pauli P. Modulation of craving by cues having differential overlap with pharmacological effect: evidence for cue approach in smokers and social drinkers. Psychopharmacology. 1999;147(3):306–13. https://doi.org/10.1007/s002130051172.

Publisher's Note

Springer Nature remains neutral with regard to jurisdictional claims in published maps and institutional affiliations.

Ready to submit your research? Choose BMC and benefit from:

- fast, convenient online submission
- thorough peer review by experienced researchers in your field
- rapid publication on acceptance
- support for research data, including large and complex data types
- gold Open Access which fosters wider collaboration and increased citations
- maximum visibility for your research: over 100M website views per year

At BMC, research is always in progress.

Learn more biomedcentral.com/submissions

BMC
